# Multi-omics reveal mechanisms of high enteral starch diet mediated colonic dysbiosis via microbiome-host interactions in young ruminant

**DOI:** 10.1186/s40168-024-01760-w

**Published:** 2024-02-24

**Authors:** Chunjia Jin, Shengru Wu, Ziqi Liang, Jun Zhang, Xinjian Lei, Hanxun Bai, Gaofeng Liang, Xiaodong Su, Xiaodong Chen, Peiyue Wang, Yue Wang, Leluo Guan, Junhu Yao

**Affiliations:** 1 College of Animal Science and Technology, Northwest A&F University, Yangling, Shaanxi 712100 People’s Republic of China; 2 Department of Agricultural, Food and Nutritional Science, University of Alberta, 116 St. and 85 Ave., Edmonton, AB T6G 2P5 Canada; 3 Key Laboratory of Livestock Biology, Northwest A&F University, Yangling, 712100 Shaanxi China

**Keywords:** Enteral starch, Colonic homeostasis, Colonic microbiome, Metabolism, Transcriptome, T_H_2 cytokine pattern

## Abstract

**Background:**

Although rumen development is crucial, hindgut undertakes a significant role in young ruminants’ physiological development. High-starch diet is usually used to accelerate rumen development for young ruminants, but always leading to the enteral starch overload and hindgut dysbiosis. However, the mechanism behind remains unclear. The combination of colonic transcriptome, colonic luminal metabolome, and metagenome together with histological analysis was conducted using a goat model, with the aim to identify the potential molecular mechanisms behind the disrupted hindgut homeostasis by overload starch in young ruminants.

**Result:**

Compared with low enteral starch diet (LES), high enteral starch diet (HES)-fed goats had significantly higher colonic pathology scores, and serum diamine oxidase activity, and meanwhile significantly decreased colonic mucosal Mucin-2 (MUC2) protein expression and fecal scores, evidencing the HES-triggered colonic systemic inflammation. The bacterial taxa *Prevotella sp. P4-67*, *Prevotella sp. PINT*, and *Bacteroides sp. CAG:927*, together with fungal taxa *Fusarium vanettenii*, *Neocallimastix californiae*, *Fusarium sp. AF-8*, *Hypoxylon sp. EC38*, and *Fusarium pseudograminearum*, and the involved microbial immune pathways including the “T cell receptor signaling pathway” were higher in the colon of HES goats. The integrated metagenome and host transcriptome analysis revealed that these taxa were associated with enhanced pathogenic ability, antigen processing and presentation, and stimulated T helper 2 cell (T_H_2)-mediated cytokine secretion functions in the colon of HES goats. Further luminal metabolomics analysis showed increased relative content of chenodeoxycholic acid (CDCA) and deoxycholic acid (DCA), and decreased the relative content of hypoxanthine in colonic digesta of HES goats. These altered metabolites contributed to enhancing the expression of T_H_2-mediated inflammatory-related cytokine secretion including GATA Binding Protein 3 (*GATA3*), *IL-5*, and *IL-13*. Using the linear mixed effect model, the variation of MUC2 biosynthesis explained by the colonic bacteria, bacterial functions, fungi, fungal functions, and metabolites were 21.92, 20.76, 19.43, 12.08, and 44.22%, respectively. The variation of pathology scores explained by the colonic bacterial functions, fungal functions, and metabolites were 15.35, 17.61, and 57.06%.

**Conclusions:**

Our findings revealed that enteral starch overload can trigger interrupted hindgut host-microbiome homeostasis that led to impaired mucosal, destroyed colonic water absorption, and T_H_2-mediated inflammatory process. Except for the colonic metabolites mostly contribute to the impaired mucosa, the nonnegligible contribution from fungi deserves more future studies focused on the fungal functions in hindgut dysbiosis of young ruminants.

Video Abstract

**Supplementary Information:**

The online version contains supplementary material available at 10.1186/s40168-024-01760-w.

## Background

During the early life, due to the dramatic nutritional and physiological changes in this period, the underdeveloped digestive tracts of young ruminants could undergo gastrointestinal dysfunction that significantly affects their growth and health. The dysfunction of gastrointestine and immunosuppression in this period leads to 14.17% of morbidity of young ruminants in China [1] and causes about 32% of deaths in the US [2], contributing to significant economic loss in the livestock industry. Thus, promoting the maturation of the digestive tract has become the major strategy to cope with this problem. At the preweaning phase, due to the immature digestive and immune system in neonatal and young ruminants, colon provides the main ecological niches for microbial colonization and fermentation [3–5] and is vulnerable to external stimuli [6–8]. Colon undertakes a significant role in young ruminants’ energy metabolism, immune system, and physiological development during this period [9, 10]. The implementation of starch to feed young ruminants to stimulate their growth and the rumen development has become a widely accepted practice. A large amount of incompletely degraded starch bypasses the rumen and flows into the hindgut, thereby triggering excessive fermentation in the hindgut [11], which is often accompanied by microbial dysbiosis, hindgut acidosis, colonic mucosal damage, and inflammation [12–14]. Although hindgut immunity plays an important role in maintaining ruminants’ health during this period which can directly influence the ruminants’ growth and performance [15, 16], the exact mechanism on its responses to high enteral starch-mediated dysbiosis has not been well-defined, preventing the effective intervention strategies.

The hindgut microbiome plays crucial roles in animal health, nutrient absorption and metabolism, gastrointestinal development, and immune function [17, 18]. Although these have been well studied in human, mice, and other monogastric animals, the understanding of immunoregulatory functions in dysbiosis in hindgut of ruminants during early life is still limited. Several studies have demonstrated the linkage between the changes in the gut microbiome and gastrointestinal dysfunction in ruminants through affecting the microbial metabolites such as short-chain fatty acids (SCFA), bile acids, and other metabolites [19, 20]. However, the majority of the host-microbiome research during early life focused on rumen development and immune functions in the small intestine. Recent study has shown that colon microbiota differs from rumen and small intestine [21], and colon microbial fermentation plays an important role in providing energy for young ruminants [22]. In addition, the colonic microbiome and transcriptome can be significantly affected by the early life feeding strategies such as delayed colostrum feeding [5, 23]. However, these studies only investigated the colonic microbiota, and/or host transcriptome of calves at 2 days of age, and lack comprehensive knowledge about long-term follow-up assessments on colonic microbial-host interactions.

In this study, we hypothesized that, in young ruminants, the enteral starch can lead to the interrupted colon homeostasis *via* affecting host-microbe interactions; by altering colonic microbiota and its metabolism, thereby affecting the colonic mucosal barrier and immune functions at gene and protein levels which could result in systemic inflammation of the young ruminants. Therefore, we assessed the host-microbe interactions by profiling colonic microbiome, metabolome, and host transcriptome using integrated omics and quantitative gene and protein expressions in young ruminants using a goat model fed with high enteral starch diet and evaluated their contributions to the host phenotypes differences including fecal scores, MUC2 biosynthesis, pathology scores, serum lipopolysaccharide (LPS) concentration, and serum diamine oxidase (DAO) activity.

## Materials and methods

### Ethics approval statement

This study was conducted in accordance with the recommendations of the Administration of Affairs Concerning Experimental Animals (Ministry of Science and Technology, China, revised in 2004). The animal using protocol was approved by the Institutional Animal Care and Use Committee of the Northwest A&F University.

### Animal experiment

The flow chart for the overall experimental design was demonstrated in the supplementary materials (Fig.S1). Forty healthy, weaned 3-month-old male goats (BW = 13.6 ± 0.23 kg) were randomly enrolled to receive either a whole corn grain diet (*n* = 20) or crushed corn grain diet (*n* = 20). Considering the processing methods of the corn affects the starch digestion in the gut, the hindgut starch content showed that the crushed corn grain diet was the high hindgut-enteral starch content (HES) group, while the whole corn grain diet was the low hindgut-enteral starch content (LES) group (Fig. S1).

All goats were housed individually in tie-stall barns and fed twice a day (0800 and 1600) with free access to water. The forage-to-concentrate ratio of the two diets was both set as 60:40 and the detailed ingredient compositions and nutrition levels of the feed are presented in Table S1. Feed samples and the dry matter intake were continually collected for 3 days every week throughout the trial. The feeding trial lasted for 90 days, and the fecal scores were evaluated for continuously 7 days (84–90 days) using a 5-grade fecal scores evaluation system that adapted from Woolsoncroft et al. [24] and Ireland-Perry and Stallings [25] (Fig.S2)﻿. The standard of this system is as follows: 5 = hard, dry pellets in a small, hard mass; 4 = hard, formable stool like pinecone that retains the shape; 3 = soft, formable stool like stone that retains its shape; 2 = soft, deformable stool that likes human normal feces; 1 = watery, liquid stool. Based on the fecal scores difference, 8 goats from each group (in total 16 goats) were selected for slaughter and sampling at the end of the trial (on day 90).

### Sample collection

Before the slaughter on day 90, blood samples were collected from jugular vein into 5-mL endotoxin free vacuum blood collection tubes and were then put into 37 °C water bath for 15 min and centrifuged at 3500 × *g* for 15 min at 4 °C for serum collection. Based on the mean values of fecal scores of two groups, eight represented LES goats and eight represented HES goats, were selected for slaughter and sampling. After slaughter, the rumen fluid samples were filtered and collected to determine the pH and SCFA content following the methods of Shen et al. [26]. The colonic digesta were collected following the procedures as described in Ye et al. [27]. Colonic digesta of each goat was mixed well and pH was assessed immediately according to the description of Liu et al. [28]. The mixed colonic digesta were then divided into three portions; (1) stored in liquid nitrogen for microbial DNA, metabolite extraction, and SCFA analysis [26, 29]; (2) processed and stored at − 20 °C for LPS analysis [27]; (3) used to analyze the nutrients’ composition [28]. For colonic tissues, about 6 cm of colonic tissue of each goat was harvested at the middle of the colon and rinsed 3 times with ice-cold sterile 0.01 M PBS buffer (pH7.2–7.4, Beijing Solarbio Science & Technology Co., Ltd, China) within 5 min of slaughter. The colonic tissue was then divided into three parts: (1) scraped using sterile slides and stored in liquid nitrogen for RNA isolation [30]; (2) fixed in 4% paraformaldehyde to make hematoxylin and eosin (H&E) staining sections for pathologic observation; (3) fixed with methanol-Carnoy’s fixative [60% (v/v) dry methanol, 30% (v/v) chloroform, 10% (v/v) glacial acetic acid] for Mucin-2 (MUC2) immunofluorescence and immunohistochemistry (IHC) detection.

### Colonic histopathology

Colonic H&E-stained sections were used for the grading of colonic inflammation as reported by Neurath et al. [31]. In brief, 0, no signs of inflammation; 1, very low level; 2, low level of leukocytic infiltration; 3, high level of leukocytic infiltration, high vascular density, thickening of the colon wall; 4, transmural infiltrations, loss of goblet cells, high vascular density, thickening of the colon wall. About 9 images per sample were taken with Nikon ECLIPSE Ni-U (Nikon Co., Ltd, Japan) at × 100 magnification, and the average scores of each sample were calculated.

### Colonic MUC2 detection using immunofluorescence and IHC staining

Immunofluorescence analysis of colon tissue was performed following the procedures described by Earle et al. [32] and Kong et al. [33]. Briefly, after deparaffinization, rehydration, and antigen retrieval, slides were blocked with bovine serum albumin for 30 min at room temperature following incubation with Anti-MUC2 antibody (EPR6145, ab134119, Abcam, UK) at 4 °C overnight. Then, slides were washed three times in PBS and incubated with secondary antibody (ZF-0311, FITC-conjugated goat anti-rabbit, ZSGB-BIO, INC, China) at 37 °C for 30 min. After incubation with the secondary antibody and DAPI, slides were washed three times in PBS, dried, and mounted with anti-fade reagent (AR1109; Boster Bioengineering, INC, China). The images were collected using FluoView FV1000 confocal laser microscope with the FV1000 software (Olympus Optical Technology Co., Ltd, Japan). IHC staining for MUC2 was performed with an initial incubation with MUC2 rabbit polyclonal (Anti-MUC2 Rabbit pAb, GB11344, Servicebio, INC, China) at a dilution of 1:100 followed by the application of the secondary biotinylated goat anti-rabbit IgG (SP-9001, ZSGB-BIO, INC, China). The slides were captured with BA400 Digital microscopic camera system (Motic China Group Co., Ltd, China) and scanned at × 400 magnification. Semi-quantitative image analysis was performed with the open-source software ImageJ, and the IHC profiler plug-in developed by Varghese et al. [34]. Software operation and data collection were performed as previously described by Alagaratnam et al. [35].

### Transcriptome analysis of colonic mucosa

Total RNA was extracted from colonic tissue using Trizol reagent (Invitrogen, Carlsbad, INC, USA). The RNA amount and purity were quantified using NanoDrop ND-1000 (NanoDrop, Wilmington, DE, INC, USA) and RNA integrity was assessed using Bioanalyzer 2100 (Agilent, INC, USA) and confirmed with denaturing agarose gel electrophoresis. The total RNAs of twelve goats (with RIN number > 7.0, 6 from each group) with differed fecal scores were subjected for transcriptome analysis using RNA-seq with the 2 × 150 bp paired-end sequencing (PE150) on an Illumina Novaseq™ 6000 (LC-Bio Technology CO., Ltd, China) according to a previous study [36]. The *capra hircus* V1 (ARS1 (GCF_001704415.1)) gene annotation list was used as the reference genome. The expression level of each transcript was calculated as the fragments per kilobase of exon per million mapped reads (FRKM) using StringTie and Ballgown [37].

### Metagenome sequencing and data processing

Total microbial DNA was extracted from colonic digesta according to the instructions of the E.Z.N.A.® Soil DNA Kit (Omega Bio-Tek, Norcross, GA, U.S.). The quality and concentration of the extracted DNA were determined by 1.0% agarose gel electrophoresis and a NanoDrop® ND-2000 spectrophotometer (Thermo Scientific, USA). The colonic digesta microbial DNAs from the same 12 goats were used for metagenome sequencing on Illumina novaseq platform (150 bp paired-end sequencing, 500-pb inserts; Illumina, INC, USA) at Majorbio Bioinformatics Technology Co. Ltd. (Shanghai, China). Fastp (https://github.com/OpenGene/fastp) was used to trim the 3′-end and 5′-endreads, and remove short (< 50 bp), low-quality (quality scores < 20), and nitrogenous bases of reads. After quality control, the reads were aligned and filtered out host sequences using BWA (http://bio-bwa.sourceforge.net; host genome: *Capra hircus* V1 (ARS1 (GCF_001704415.1) [38]. The filtered reads were then de novo assembled using Megahit (https://github.com/voutcn/megahit) [39] and open reading frames (ORFs) were predicted using MetaGene (http://metagene.cb.k.u-tokyo.ac.jp/) from the contigs with the length > 300 bp [40]. Assembled contigs were clustered into a nonredundant dataset using CD-HIT (95% identity; http://www.bioinformatics.org/cd-hit/) [41]. Then, SOAPaligner (95% identity; http://soap.genomics.org.cn/) was used to map and calculate the abundances of microbial genes [42]. Taxonomic assessment of colonic microbiome was performed using DIAMOND (https://github.com/bbuchfink/diamond) [43] based on NCBI nonredundant (NCBI-NR) database. Metagenome functions were annotated using DIAMOND via KEGG (*E*-value ≤ 1e − 5) [44]. The carbohydrate-active enzyme (CAZymes) annotation was performed using hmmscan (*E*-value, 1e − 5; http://hmmer.org/; Edgar, 2010) based on the carbohydrate-active enzyme database (http://www.cazy.org/) [45].

### Validation of differentially expressed genes using qPCR analysis

Colonic total RNA of all slaughtered and sampled goats was reverse transcribed using Prime Script™ RT Master Mix (Takara, INC, China). The targeted genes include *VSIG1*, *CDH26, CLDN4, KIR3DL, SPOCK1, TRAV, MASP1, C4BPA, c-Maf*, and *A1AT1* identified from RNA-seq*, Claudin1*, *Claudin4*, *Occludin*, and *ZO-1* for barrier function-related genes, *IL-1B*, *IL-6*, *IL-10*, *IL-12*, *IL-22*, *IFN-γ*, and *TNF-α*, for pro-inflammatory-related genes, *IL-2*, *IL-5*, *IL-13*, *IL-14*, *GATA3*, *CMIP*, and *TBX21* for T_H_2-related genes, and *NHE1*, *NHE2*, *NHE3*, *AQP1*, *AQP3*, *CFTR*, and *CLCN2* for water and ion exchange related genes. qPCR was conducted on iCycler IQTM5 (Bio-Rad, INC, USA) using SYBR green dye (TB Green® Premix Ex Taq™ II Tli RNaseH Plus, Takara, China) following the standard program [46]. The data of the gene expression were normalized to the housekeeping gene (*β-actin*) using the 2^−ΔΔCT^ method [47]. The primers and amplicon sizes of genes are shown in Table S2.

### Metabolomic analysis of colonic digesta

The same 12 colon digesta were thawed at 4 °C, and 1000 μL methanol/ acetonitrile/ H_2_O (2:2:1, v/v/v) were added to homogenized solution for metabolite extraction. Then they were re-dissolved in 100 μL acetonitrile/ water (1:1, v/v) solvent and subjected to LC–MS analysis using an UHPLC (1290 Infinity LC, Agilent Technologies, INC, Germany), coupled to a quadrupole time-of-flight (AB Sciex TripleTOF 6600) in Shanghai Applied Protein Technology Co., Ltd. The raw MS data (wiff.scan files) were converted to MzXML files using ProteoWizard MSConvert before importing into freely available XCMS software [48]. For peak picking, the following parameters were used: centWave m/z = 25 ppm, peakwidth = c (10, 60), prefilter = c (10, 100). For peak grouping, bw = 5, mzwid = 0.025, minfrac = 0.5 were used. CAMERA (Collection of Algorithms of Metabolite Profile Annotation) [49] was used for annotation of adducts. In the extracted ion features, only the variables having more than 50% of the nonzero measurement values in at least one group were kept. Compound identification of metabolites was performed by comparing of accuracy m/z value (< 25 ppm), and MS/MS spectra with a published database established with available authentic standards [50, 51]. After normalized to total peak intensity, the processed data were analyzed by R package ropls [53], where it was subjected to multivariate data analysis, including Pareto-scaled principal component analysis (PCA) and orthogonal partial least squares discriminant analysis (OPLS-DA). According to Li et al. [53], the seven-fold cross-validation and response permutation testing were used to evaluate the robustness of the model. The variable importance in the projection (VIP) value of each variable in the OPLS-DA model was calculated to indicate its contribution to the classification. Origin assessment of colonic metabolites was based on MetOrigin database (2022–08 version, *Capra hircus*, https://metorigin.met-bioinformatics.cn) [53].

### Analysis of LPS concentration and DAO activity

LPS of colonic digesta and serum was detected using Bioendo EC Endotoxin Test Kit (End-point Chromogenic Assay, Xiamen Bioendo Technology Co., Ltd, China) following the manufacturer’s procedure and as reported by Chen et al. [53]. The concentration of digesta and serum LPS was calculated using the standard curve and expressed as EU/g and EU/mL, respectively. Serum DAO activity was measured with DAO assay kit (A088-1–1, Njjcbio, INC, China) with ultraviolet chromatometry. The serum DAO activity was expressed as U/L. All measurements were conducted with three replicates.

### Omics-based explainability of phenotype variations

The variation in host phenotypes that could be explained by the colonic microbial variance, metabolic variance, and KOs variance were further evaluated using the methods described in Xue et al. [53]. Colonic microbiome, metabolites, and KOs were normalized to construct the relationship matrix, respectively [53]. The liner mixed model (LMM) was conducted as *y*_*ij*_ = *μ* + starch_*i*_ + *x*_*j*_ + *e*_*ij*_ with lme4 package in R [53]. *y*_*ijk*_ is the fecal scores/ MUC2 expression/ pathology scores/ serum LPS; *μ* is the model intercept; starch_*i*_ is the fixed effect; *x*_*j*_ is the colonic bacteria/bacterial functions/ fungi/ fungal functions/ metabolites random effect for the *j*th animal∼NID (0, Xσ^2^_X_), σ^2^_X_ is the colonic bacteria/ bacterial functions/ fungi/ fungal functions/ metabolites variance. The X is the colonic bacteria/ bacterial functions/ fungi/ fungal functions/ metabolites relationship matrix; and *e*_*ij*_ is the residual effects.

### Statistical analysis

Except for the omics data, dry matter intake (DMI), digesta nutrients’ compositions, pH, SCFAs, fecal scores, histopathology scores, MUC2 IHC assessment, LPS, DAO, and the mRNA relative abundance (qPCR) were analyzed by using Grubbs’ test and assessed by the independent sample *T*-test in SPSS 20.0 (SPSS INC, USA). For the omics data, *P* value was corrected by using the false discovery rate method (FDR) [53]. For host transcriptome, the differentially expressed genes (DEGs) were determined with fold change ≥ 2 or ≤ 0.5 and FDR < 0.05 using DESeq2 in R package [33, 53], and then DEGs were subjected to KEGG enrichment analysis. 0.05 < FDR < 0.1 was referred to as approaching significance.

Based on metagenomics data, the microbial taxa that were present in more than 60% of the samples for either group were retained for downstream analysis. Colonic bacterial and fungal species were compared by LEfSe with LDA > 2 and *P* < 0.05 [53]. Abundances of microbial functions, including, but not limited to, KEGG Orthology (KO) terms, pathways, and CAZymes were normalized into reads per kilobase million (RPKM) in at least 60% of the samples for either group. The differentially functional genes were selected by using LEfSe methods (LDA > 2, *P* < 0.05). Metabolomics data was log2 transformed [53]. Metabolites with the VIP > 1 were further applied to *T*-test at univariate level to measure the significance of each metabolite. The *FDR* < 0.05 was considered as statistically significant. Considering the high diversity, high dimensionality, and discrete distribution of omics data, we used the Spearman correlation test in the present study to explore the relationships among the phenotypes, nutrients, microbiome, metabolites, and host transcripts. |R|> 0.5, *P* < 0.05 was used to identify significant correlations. Cytoscape 3.6.1 was used to construct the network.

## Results

### Enteral starch altered the phenotypes of hindgut in goats

**Fig. 1:**
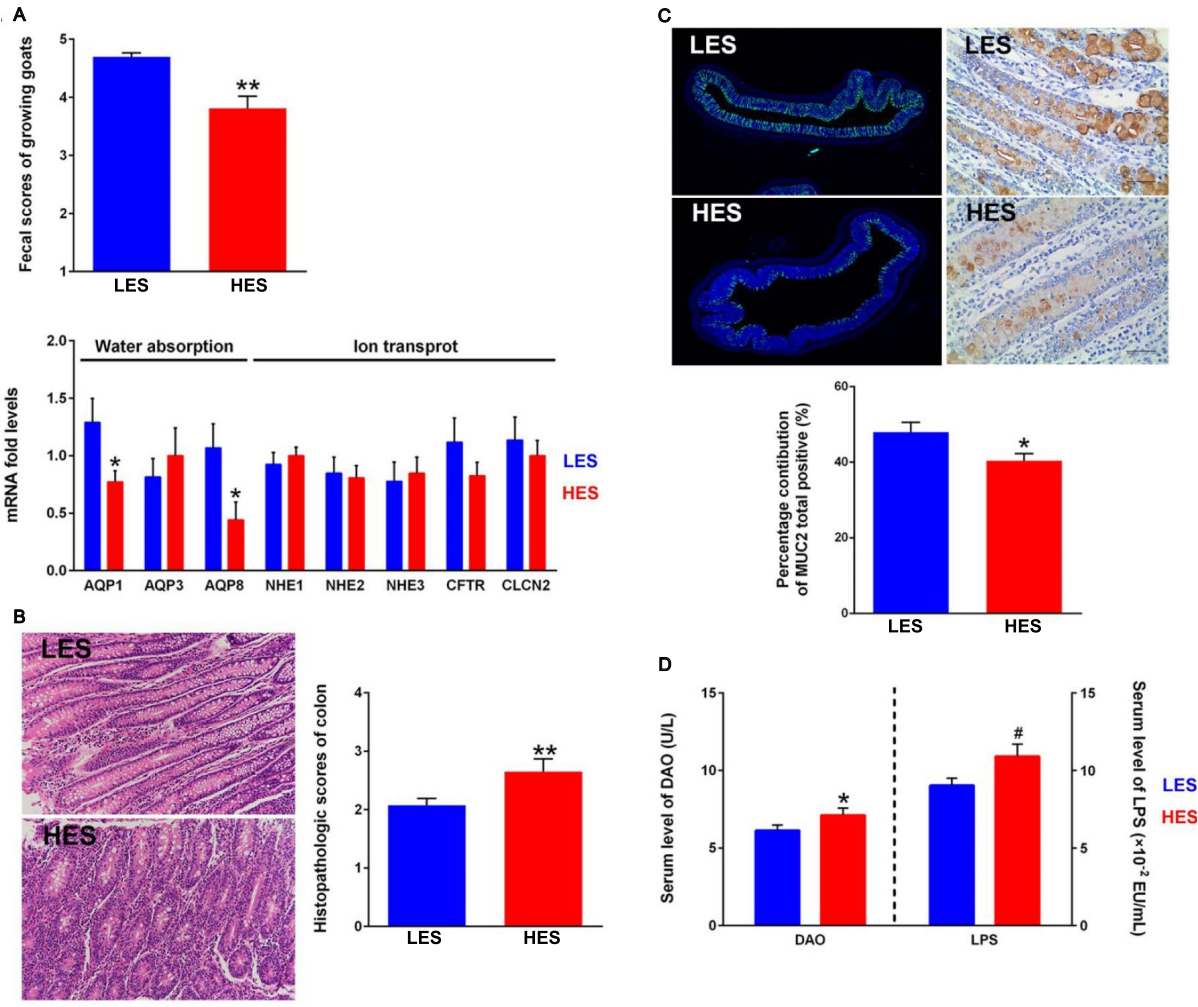
Host phenotypes and related gene expression of two groups of goats fed with low enteral starch (LES) and high enteral starch (HES) diets. **A** Fecal scores (*n* = 20) and gene expression of water absorption and ion transport (*n* = 8) of two groups. **B** Representative H&E-stained colonic sections and colonic histopathology scores of two groups (*n* = 8). **C** Representative colonic MUC2 immunofluorescence and IHC sections with semi-quantitative analysis of MUC2 biosynthesis in colonic mucosa (%) of two groups (*n* = 8). For immunofluorescence sections, samples were stained with DAPI (blue) and MUC2 antibody (green). For IHC sections, brown staining shows MUC2 expression, hematoxylin (blue) was used as a counterstain for nuclei. **D** Serum level of LPS and DAO between two groups of growing goats. The difference between two groups was identified by independent sample *T*-test. Symbols indicate significance (∗ ∗ , *P* < 0.01; ∗ , *P* < 0.05; #, 0.05 < *P* < 0.1)

Primarily, through the fecal assessment of 40 goats, we found that HES significantly decreased fecal scores of goats (*P* = 0.001; Fig. 1A). Based on the mean values of fecal scores of two groups, eight LES goats (BW = 19.77 ± 2.73 kg, fecal scores = 4.42 ± 0.13) and eight HES goats (BW = 17.69 ± 5.55 kg, fecal scores = 3.42 ± 0.30) were selected for slaughter and sampling. The starch content of post-rumen digesta (0.05 < *P* < 0.1, Fig.S3A), colonic digesta (0.05 < *P* < 0.1, Fig.S3B and C), and colonic pathology scores (*P* < 0.05; Fig. 1B) were higher in the HES goats. Immunofluorescence and IHC results showed the lower MUC2 protein expression in the HES goats (Fig. 1C). Further, the serum level of DAO (*P* < 0.05) and LPS (0.05 < *P* < 0.1) in the HES goats were higher than those in LES goats (Fig. 1D). Moreover, no significant differences in the ruminal pH, total SCFA concentration, and proportion of acetate, propionate, and butyrate were identified between the two groups (*P* > 0.05; Table S3).

### Metagenome analysis revealed HES led changes in the colonic microbiome and its functions

**Fig. 2:**
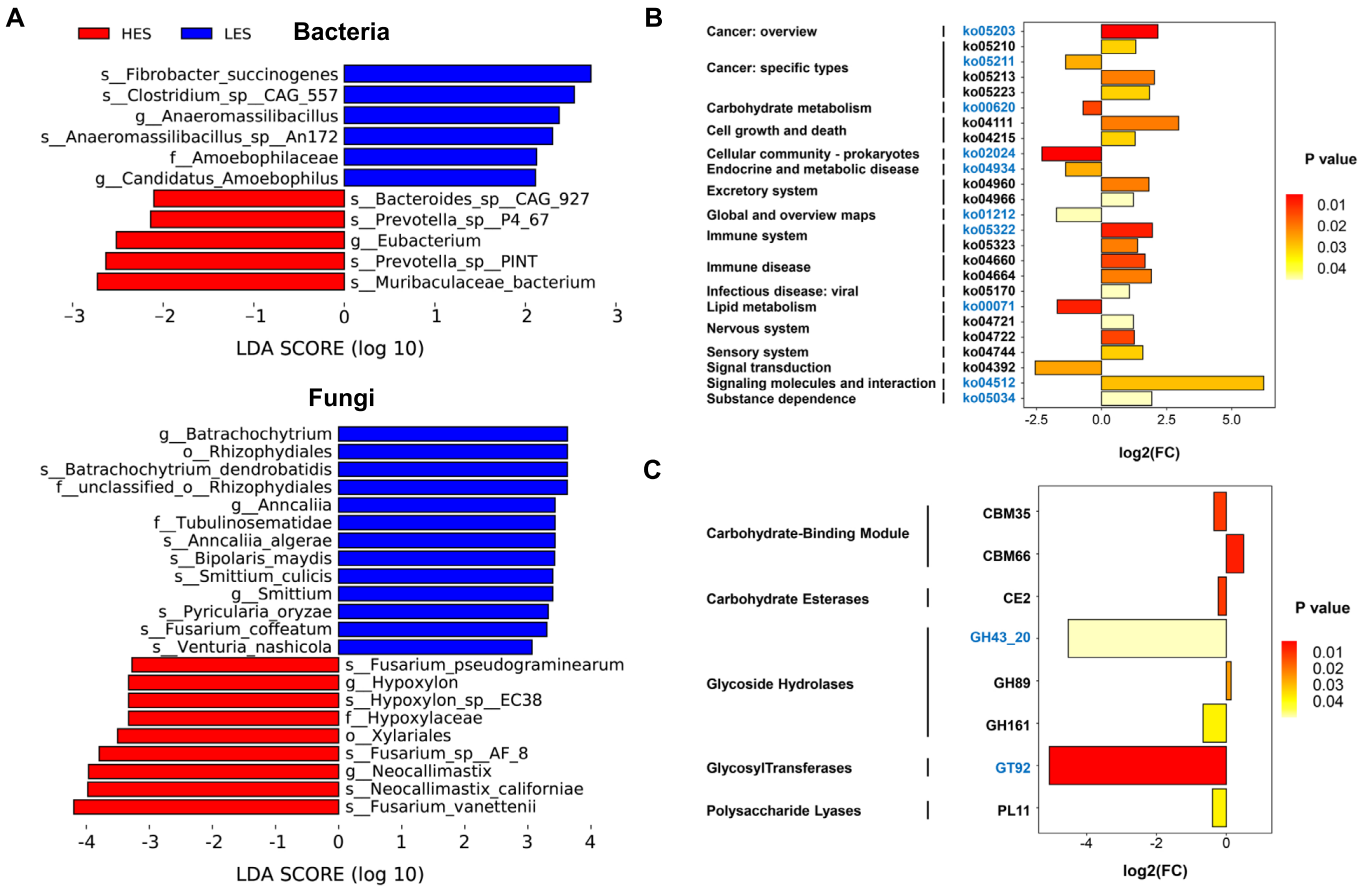
Effects of enteral starch content on colonic microbiota and microbial functions (*n* = 6). **A** Differential colonic bacterial and fungal species between LES and HES groups. Significant differences were identified by LEfSe analysis with LDA > 2, *P* < 0.05. **B** HES/LES fold change of significantly enriched KEGG pathways (level 3). FC = HES/LES. ko05203, Viral carcinogenesis; ko05210, Colorectal cancer; ko05211, Renal cell carcinoma; ko05213, Endometrial cancer; ko05223, Non-small cell lung cancer; ko00620, Pyruvate metabolism; ko04111, Cell cycle—yeast; ko04215, Apoptosis—multiple species; ko02024,Quorum sensing; ko04934, Cushing syndrome; ko04960, Aldosterone-regulated sodium reabsorption; ko04966, Collecting duct acid secretion; ko01212, Fatty acid metabolism; ko05322, Systemic lupus erythematosus; ko05323, Rheumatoid arthritis; ko04660, T cell receptor signaling pathway; ko04664, Fc epsilon RI signaling pathway; ko05170, Human immunodeficiency virus 1 infection; ko00071, Fatty acid degradation; ko04721, Synaptic vesicle cycle; ko04722, Neurotrophin signaling pathway; ko04744, Phototransduction; ko04392, Hippo signaling pathway—multiple species; ko04512, ECM-receptor interaction; ko05034, Alcoholism. The black font color indicated bacterial function, and the blue font color indicated fungal function. **C** HES/LES fold change of significantly enriched CAZymes. FC = HES/LES. The black font color indicated bacterial function, and the blue font color indicated fungal function

About 5–6 Gb per sample of metagenome sequencing data from 12 goats were obtained. After quality control, removing host genes, and de novo assembly, a total of 5,980,331 contigs were obtained (the N50 length of 672 ± 15 bp), with 498,361 ± 19,272 per sample. At the bacterial species level, three species, including *Fibrobacter succinogenes*, *Clostridium sp. CAG:557*, and *Anaeromassilibacillus sp. An172* were reduced in HES goats (LDA > 2, *P* < 0.05), while *Bacteroides sp. CAG:927*, *Prevotella sp. PINT*, *Prevotella sp. P4-67*, and *Muribaculaceae bacterium* were enriched in the HES goats (LDA > 2, *P* < 0.05; Fig. 2A). At the fungal species level, several species, including *Batrachochytrium dendrobatidis*, *Anncaliia algerae*, *Bipolaris maydis*, *Smittium culicis*, *Fusarium coffeatum*, *Pyricularia oryzae*, and *Venturia nashicola* were reduced in HES goats (LDA > 2, *P* < 0.05), while *Fusarium vanettenii*, *Neocallimastix californiae*, *Fusarium sp. AF-8*, *Hypoxylon sp. EC38*, and *Fusarium pseudograminearum* were increased in the HES goats (LDA > 2, *P* < 0.05; Fig. 2A).

The functions of the colonic bacteria and fungi using KEGG enrichment analysis revealed 333 KEGG pathways were identified and belonged to 6 KEGG level 1 categories, including 140 pathways for Metabolism, 67 pathways for Human Diseases, 54 pathways for Organismal Systems, 27 pathways for Cellular Processes, 24 pathways for Environmental Information Processing, and 20 pathways for Genetic Information Processing. As shown in Fig. 2B, 8 pathways of bacteria and 10 pathways of fungi were enriched in KEGG level 3 categories in the colon microbiome of HES fed goats. Additionally, 503 genes encoding CAZymes were identified, including 16 auxiliary activities (AA), 64 carbohydrate-binding modules (CBM), 15 carbohydrate esterases (CE), 231 glycoside hydrolases (GH), 72 glycosyltransferases (GT), and 62 polysaccharide lyases (PL). Among these genes, 1 CBM (CBM35),1 CE (CE2), 2 GH (GH43_20 and GH161), 1 GT (GT92), and 1 PL (PL11) were reduced and 1 CBM (CBM66) and 1 GH (GH89) were increased in the colon of HES goats (Fig. 2C).

### Enteral starch also altered metabolism in the colon

**Fig. 3:**
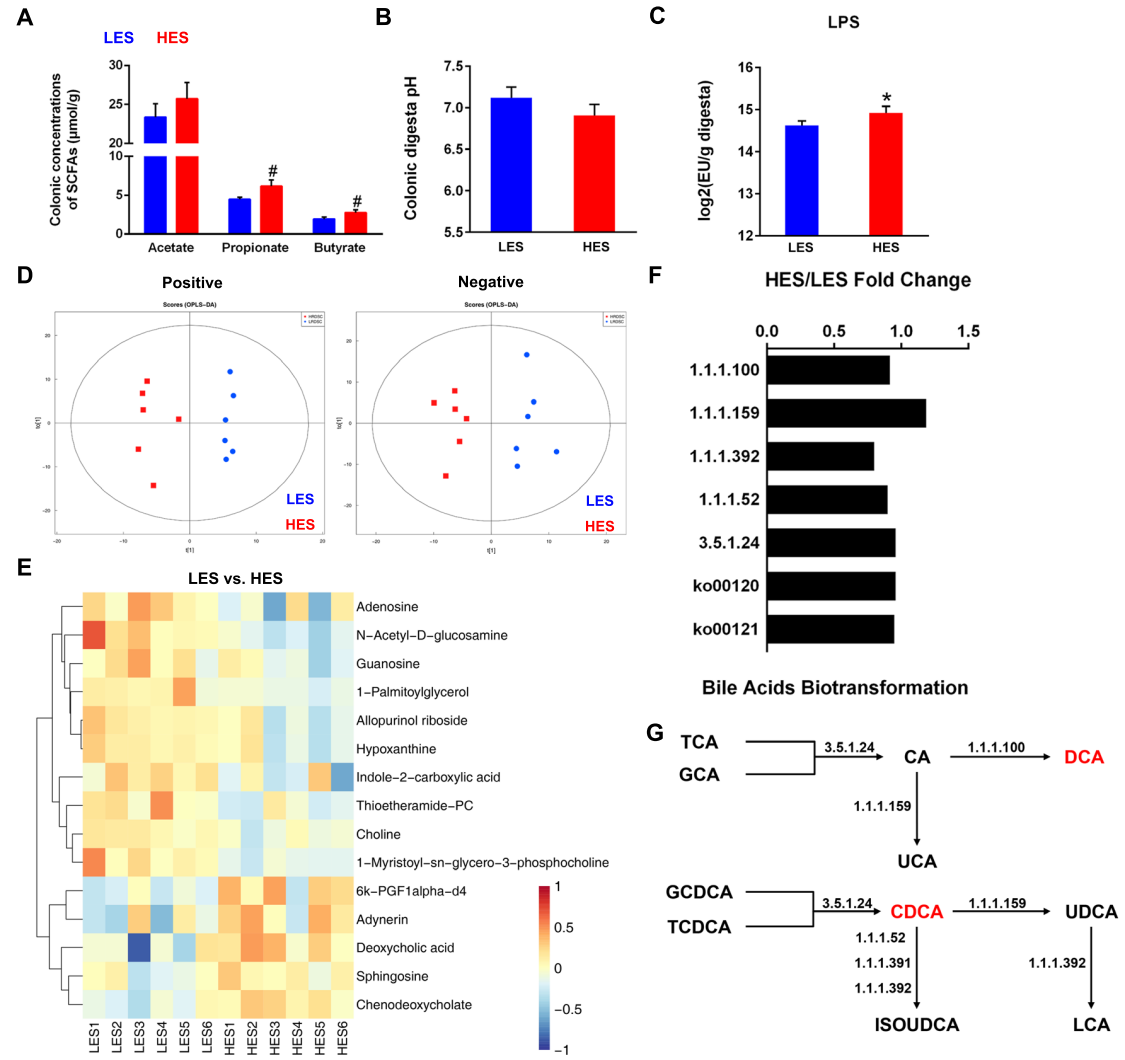
Effects of enteral starch on colonic substrate metabolism (*n* = 8 for SCFAs, pH, and LPS, and *n* = 6 for metabolome). **A** Concentrations of SCFAs in colonic digesta (μM/g). The difference between two groups was identified by independent sample *T*-test. **B** Colonic digesta pH of two groups of growing goats. The difference between two groups was identified by independent sample *T*-test. **C** Absolute concentration of LPS in colonic digesta on log2-transformed concentration correcting for subject. The difference between two groups was identified by independent sample *T*-test. **D** OPLS-DA analysis of colonic substrate metabolism. **E** Heatmap of 15 differential metabolites in colonic digesta (VIP > 1, 0.05 < *P* < 0.1; both negative and positive modes). The upregulated metabolites are shown in red, whereas the downregulated metabolites are presented in blue. **F** Microbial KO terms related to the bile acid microbial biotransformation of the HES goats compared with the LES goats. The values are presented as HES/LES fold change. Mann–Whitney *U* test was carried out for comparing the two groups. EC: 1.1.1.100, K00059, 3-oxoacyl-[acyl-carrier-protein] reductase; EC: 1.1.1.159, K00076, 7-alpha-hydroxysteroid dehydrogenase; EC: 3.5.1.24, K01442, choloylglycine hydrolase; EC: 1.1.1.52, K22604, 3alpha-hydroxycholanate dehydrogenase (NAD +); EC: 1.1.1.392, K22605, 3alpha-hydroxycholanate dehydrogenase (NADP +); EC: 1.1.1.391, K22606, 3beta-hydroxycholanate 3-dehydrogenase (NAD +); ko00120, Primary bile acid biosynthesis; ko00121, Secondary bile acid biosynthesis. **G** Microbial biotransformation of the bile acids in the colon of HES goats. Red: upregulation. EC: 1.1.1.100, K00059, 3-oxoacyl-[acyl-carrier-protein] reductase; EC: 1.1.1.159, K00076, 7-alpha-hydroxysteroid dehydrogenase; EC: 3.5.1.24, K01442, choloylglycine hydrolase; EC: 1.1.1.52, K22604, 3alpha-hydroxycholanate dehydrogenase (NAD +); EC: 1.1.1.392, K22605, 3alpha-hydroxycholanate dehydrogenase (NADP +); EC: 1.1.1.391, K22606, 3beta-hydroxycholanate 3-dehydrogenase (NAD +). Symbols indicate significance (∗ , *P* < 0.05; #, 0.05 < *P* < 0.1)

The colonic concentrations of SCFAs [especially propionate (0.05 < *P* < 0.1) and butyrate (0.05 < *P* < 0.1)] had increasing trends in HES goats (Fig. 3A), although the pH value of colonic digesta was comparable between the two groups (Fig. 3B). We also found higher colonic concentration of LPS in the HES goats (*P* < 0.05; Fig. 3C). Metabolomic analysis of colonic digesta revealed a total of 554 annotated metabolites with distinguished metabolome profiles between the HES and LES groups based on both the partial least squares of discriminant analysis and orthogonal partial least squares of discriminant analysis (Fig. 3D). Based on MetOrigin database, 296 metabolites were identified and found 2 host metabolites, 77 microbial metabolites, and 172 co-metabolites (Table S4). Less abundance of choline (co-metabolite, *P* < 0.05), allopurinol riboside (food related metabolite, *P* < 0.05), hypoxanthine (co-metabolite, *P* < 0.05), 1-myristoyl-sn-glycero-3-phosphocholine (unknown origin, *P* < 0.05), 1-palmitoylglycerol (unknown origin, *P* < 0.05), N-acetyl-d-glucosamine (co-metabolite, *P* < 0.05), adenosine (co-metabolite, 0.05 < *P* < 0.1), thioetheramide-PC (unknown origin, 0.05 < *P* < 0.1), indole-2-carboxylic acid (unknown origin, 0.05 < *P* < 0.1), and guanosine (co-metabolite, 0.05 < *P* < 0.1), as well as more abundance of adynerin (unknown origin, 0.05 < *P* < 0.1), sphingosine (co-metabolite, 0.05 < *P* < 0.1), chenodeoxycholic acid (host metabolite, CDCA, *P* < 0.05), 6 k-PGF1α-d4 (unknown origin, *P* < 0.05), and deoxycholic acid (food related metabolite, DCA, *P* < 0.05) were identified in the HES goats (Fig. 3E). Further, microbial functions (KO terms) based on the metagenome data that related to the bile acid (BA) metabolism were inspected to explore the microbial biotransformation of BAs in the colon (Fig. 3F and G). However, no differences were observed in ko00120 (Primary bile acid biosynthesis), ko00121 (Secondary bile acid biosynthesis), and KO terms related to the BA metabolism between the two groups. These results indicated the increased DCA and CDCA may be induced by their accumulation rather than enhancing microbial biotransformation of BAs.

### Enteral starch altered the mucosal transcriptome in the colon

**Fig. 4:**
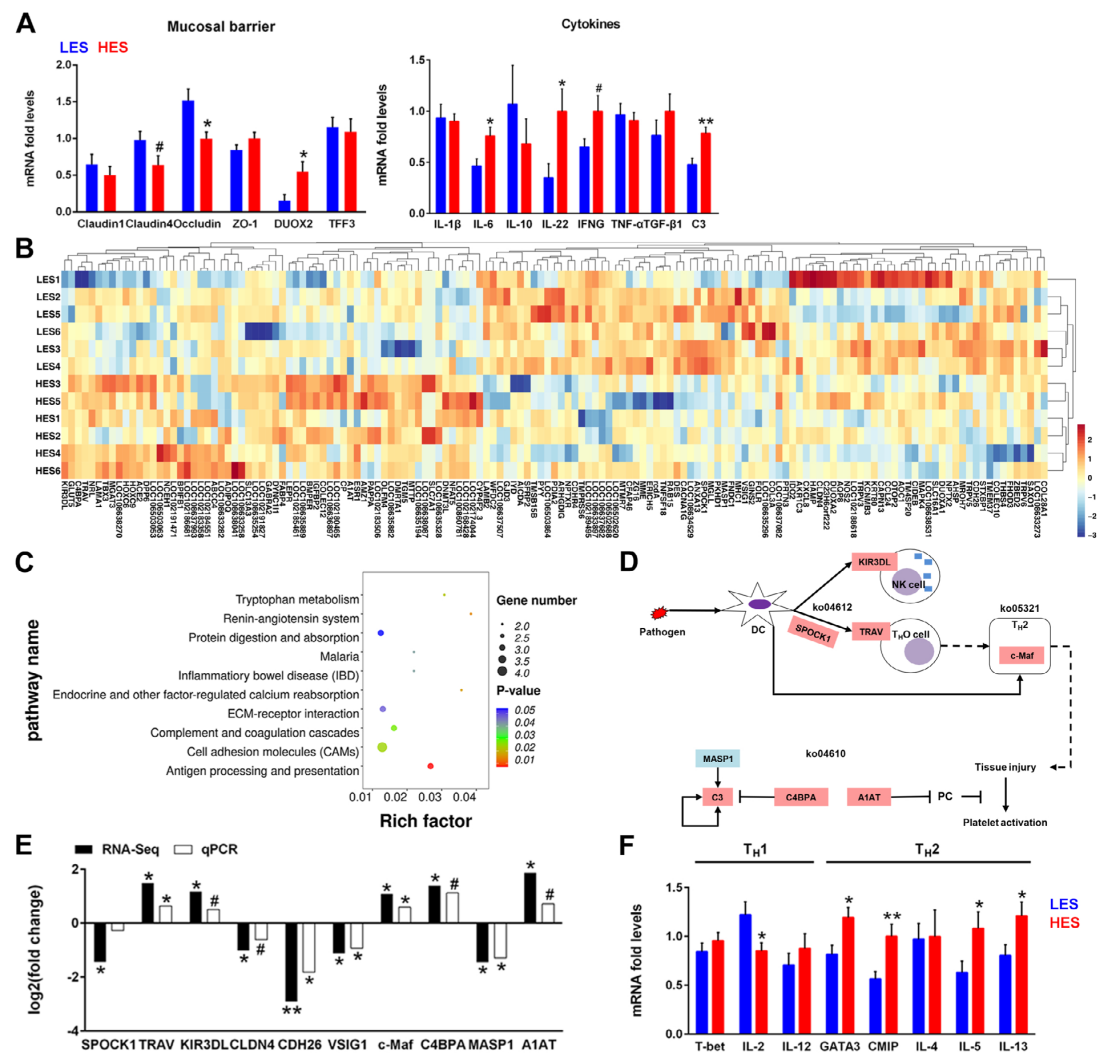
Effects of enteral starch content on colonic mucosal barrier and transcriptional profile (*n* = 8 for mucosal defense parameters, and *n* = 6 for host transcriptome). **A** Gene expression of colonic mucosal defense of two groups of growing goats. The difference between two groups was identified by independent sample *T*-test. **B** Heatmap of colonic mucosal DEGs. Compared with LES, 64 DEGs were upregulated and 81 DEGs were downregulated significantly in HES goats, respectively. **C** KEGG pathway enrichment analysis of DEGs (top10). **D** DEGs related to differential pathways in the colon of HES goats (ko04610, ko04612, and ko05321). ko04610, Complement and coagulation cascades; ko04612, Antigen processing and presentation; ko05321, Inflammatory bowel disease (IBD). The upregulated DEGs are shown in red, whereas the downregulated DEGs are presented in blue. **E** DEGs (RNA-Seq) related to the mucosal immunologic pathways of the HES goats compared with the LES goats (the difference was identified by Mann–Whitney *U* test.). qPCR validation of DEGs in the colonic mucosa of HES goats compared with the LES goats (the difference was identified by independent sample *T*-test). The values are presented as log_2_ (fold change). **F** Effects of enteral starch on the gene expression which involved in colonic mucosal T helper (T_H_) cells’ function. The difference between two groups was identified by independent sample *T*-test. Symbols indicate significance (∗ ∗ , *P* < 0.01; ∗ , *P* < 0.05; #, 0.05 < *P* < 0.1)

Further transcriptome profiling revealed a total of 27,137 genes expressed and among them 145 were differentially expressed genes (DEGs, fold change (FC) ≥ 2 or ≤ 0.5, *P* < 0.05) identified in colonic mucosa with 64 DEGs upregulated and 81 DEGs downregulated in HES goats when compared to those in LES group (Fig. 4B). Further analysis of KEGG pathway of the DEGs identified several functional categories (Fig. 4C). The enriched top 10 pathways in HES group included antigen processing and presentation (*P* < 0.05), renin-angiotensin system (*P* < 0.05), endocrine and other factor-regulated calcium reabsorption (*P* < 0.05), tryptophan metabolism (*P* < 0.05), cell adhesion molecules (CAMs, *P* < 0.05), complement and coagulation cascades (*P* < 0.05), inflammatory bowel disease (IBD, *P* < 0.05), malaria (*P* < 0.05), ECM-receptor interaction (*P* < 0.05), and protein digestion and absorption (0.05 < *P* < 0.1).

Moreover, KEGG analysis revealed that 10 DEGs were mostly enriched in the immune-associated pathways and the expression of these DEGs was validated by qPCR (Fig. 4E). T cell receptor alpha chain V region (*TRAV*), transcription factor Maf (*c-Maf*), and alpha-1-antitrypsin (*A1AT*) were significantly upregulated in the HES goats (*P* < 0.05). Cadherin26 (*CDH26*), v-set and immunoglobulin domain containing 1 (*VSIG1*), and mannan-binding lectin serine protease 1 (*MASP1*) were significantly downregulated in HES goats (*P* < 0.05). The expression of killer cell immunoglobulin-like receptor 3DL (*KIR3DL*), complement component 4 binding protein alpha (*C4BPA*), and Claudin 4 (*CLDN4*) showed the same trends with the transcriptome results (0.05 < *P* < 0.1). Besides, considering the upregulating of CDCA and DCA in the colon of HES goats, we inspected colonic bile acid receptors and found no difference in the gene expression of apical sodium-dependent bile acid transporter (*ABST*), ATP binding cassette subfamily C member 3 (*ABCC3*), and organic solute transporter alpha–beta (*OSTα-OSTβ*; Fig.S4).

Due to the upregulation of *c-Maf* in the colon of HES goats, we further detected the expression levels of key genes in colonic mucosa that are associated with T_H_1 and T_H_2 cells (Fig. 4F) and found that the expression of *IL-2* of T_H_1 cells was significantly downregulated in the HES goats (*P* < 0.05). The expression of the master transcription factor *GATA3*, *c-Maf* inducing protein (*CMIP*), *IL-5*, and *IL-13* of T_H_2 cells were significantly upregulated in the HES goats (*P* < 0.05). Similarly, the expression of mucosal barrier and cytokine genes was evaluated using qPCR (Fig. 4A). The results showed upregulated *DUOX2* (*P* < 0.05) and downregulated *Claudin4* (0.05 < *P* < 0.1) and *Occludin* (*P* < 0.05) in the colon of HES goats. HES increased the expression of *IL-6* (*P* < 0.05), *IL-22* (*P* < 0.05), *IFN-γ* (0.05 < *P* < 0.1), and *C3* (*P* < 0.01) in colonic mucosa. Besides, we also found that HES decreased the expression of *AQP1* (*P* < 0.05) and *AQP8* (*P* < 0.05) (Fig. 1A).

### Multi-omics integration analysis and phenotypic omics contribution assessment

Connections between phenotypes, microbial species, functions, metabolites, and/or host gene expression were determined by performing an association study (using Spearman correlation coefficient). Firstly, Spearman’s correlations among the enteral nutrients, phenotypes, microbes, and microbial functions were shown in Fig.S5. *Bacteroides sp. CAG:927*, *Fusarium pseudograminearum*, and *Neocallimastix californiae* showed positive correlations with the content of starch, while *Prevotella sp. P4-67* and *Bacteroides sp. CAG:927* were positively related to pathology scores (R > 0.50, *P* < 0.05; Fig.S5A). Changes in MUC2 biosynthesis and pathology scores were closely related to bacterial functions (|R|> 0.50, *P* < 0.05; Fig.S5B).

**Fig. 5:**
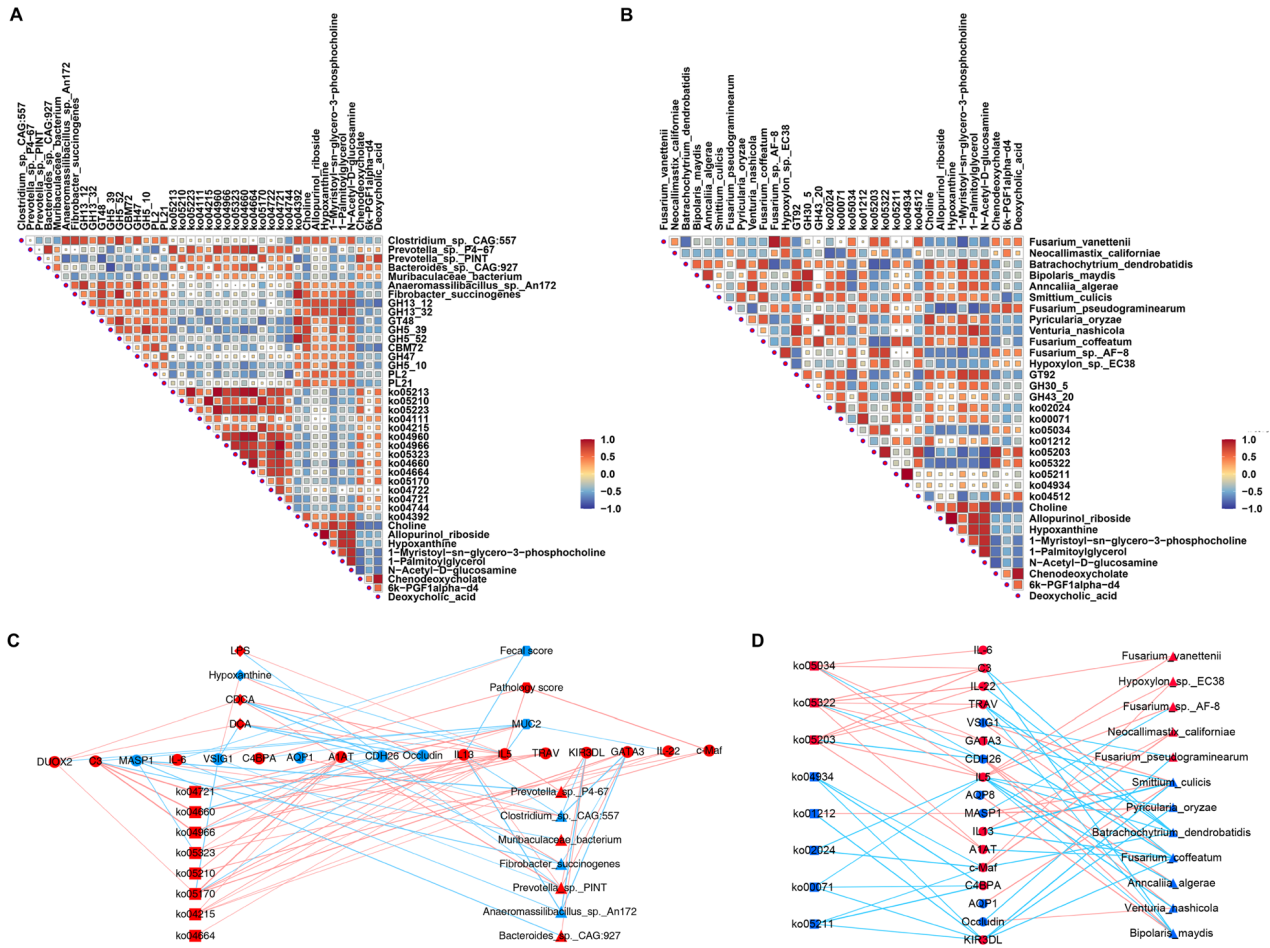
Multi-omics integration included host phenotypes (fecal scores, MUC2 biosynthesis, and pathology scores), colonic microbiome, microbial functions, metabolome, and host transcriptome. **A** Spearman correlation among the metabolites, bacteria, and bacterial functions. **B** Spearman correlation among the metabolites, fungi, and fungal functions. **C** Spearman correlation network among the host phenotypes, host DEGs, metabolites, bacteria, and bacterial functions. The width of edges is proportional to the correlation strength. The color of edges: red, positive; blue, negative. The color of nodes: red, significantly enriched in HES; blue: significantly enriched in LES. The shape of nodes: ellipse, DEGs; diamond, metabolites; hexagon, phenotypes; rectangle, microbial functions; triangle, microbiota. Only strong correlations were displayed (|R|> 0.5, *P* < 0.05). **D** Spearman correlation network among the host DEGs, fungi, and fungal functions. The width of edges is proportional to the correlation strength. The color of edges: red, positive; blue, negative. The color of nodes: red, significantly enriched in HES; blue: significantly enriched in LES. The shape of nodes: ellipse, DEGs; rectangle, microbial functions; triangle, microbiota. Only strong correlations were displayed (|R|> 0.5, *P* < 0.05)

Ninety-two significant relationships were discovered among the metabolites, bacteria, and bacterial functions (|R|> 0.50, *P* < 0.05; Fig. 5A). Positive correlations existed between the 5 bacterial species (*Anaeromassilibacillus sp. An172*, *Bacteroides sp. CAG:927*, *Clostridium sp. CAG:557*, *Prevotella sp. P4-67*, and *Prevotella sp. PINT*) and ko04664, ko05323, ko05170, and GH47 (0.65 < R < 0.80, *P* < 0.05), while negative correlations existed with ko05170, GH5_39, and GH47 (− 0.87 < R < − 0.66, *P* < 0.05), respectively. *Fibrobacter succinogenes* and *Muribaculaceae bacterium* also exhibited correlations with ko05170, ko05323, GH47, and PL21 (0.50 <|R|< 0.65, *P* < 0.05), respectively. Besides, 94 significant relationships among the metabolites, fungi, and fungal functions were identified by Spearman’s correlations analysis (|R|> 0.50, *P* < 0.05; Fig. 5B). Positive correlations existed between the 2 fungal species (*Fusarium vanettenii* and *Fusarium sp. AF-8*) and ko05203 and ko05322 (0.60 < R < 0.75, *P* < 0.05), respectively. We also found that CDCA and DCA have positive correlations with *Prevotella sp. PINT* and negative correlations with *Venturia nashicola* and *Fusarium coffeatum*.

We also observed associations among host phenotypes, DEGs, luminal metabolites, bacteria, and bacterial functions in Fig. 5C (|R|> 0.50, *P* < 0.05). Pathology and fecal scores were significantly correlated with the expression of T_H_2 cytokine pattern. Spearman’s correlations between the phenotypes and metabolites revealed negative correlations between the MUC2 biosynthesis and CDCA and DCA (− 0.87 < R < − 0.74, *P* < 0.05). CDCA, DCA, and LPS showed the negative correlations with the gene expression of CAMs (*CDH26* and *VSIG1*) and epithelial tight junction (*Occludin*). *Prevotella sp. P4-67*, *Prevotella sp. PINT*, and *Bacteroides sp. CAG:927* greatly affected the expression of *c-Maf*, *GATA3*, and *IL-5* that involved in the T_H_2-mediated cytokine pattern. *Fibrobacter succinogenes* and *Anaeromassilibacillus sp. An172* showed the capacity of maintaining the colonic homeostasis by inhibiting the gene expression of antigen processing and presentation (*TRAV* and *KIR3DL*) and T_H_2-mediated cytokine pattern (*GATA3*, *IL-5*), respectively. Other significant correlations, such as the positive correlations between the bacterial functions (ko04721, ko04660, ko04966, ko05323, ko05210, ko05170, ko04215, and ko04664) and host immune pathway-related gene expression, were also observed (|R|> 0.50, *P* < 0.05). Besides, *Fusarium vanettenii*, *Fusarium sp. AF-8*, *Hypoxylon sp. EC38*, *Neocallimastix californiae*, ko05034, ko05203, and ko05322 were associated with the T_H_2-related gene expression of *c-Maf*, *GATA3*, *IL-5*, and *IL-13*, respectively (|R|> 0.50, *P* < 0.05; Fig. 5D). *Batrachochytrium dendrobatidis*, *Bipolaris maydis*, *Fusarium coffeatum*, *Smittium culicis*, *Pyricularia oryzae*, and *Anncaliia algerae* showed the negative correlations with the gene expression of antigen processing and presentation (*TRAV* and *KIR3DL*) and T_H_2-mediated cytokine pattern (*GATA3*, *IL-5*), respectively (|R|> 0.50, *P* < 0.05; Fig. 5D).

**Fig. 6:**
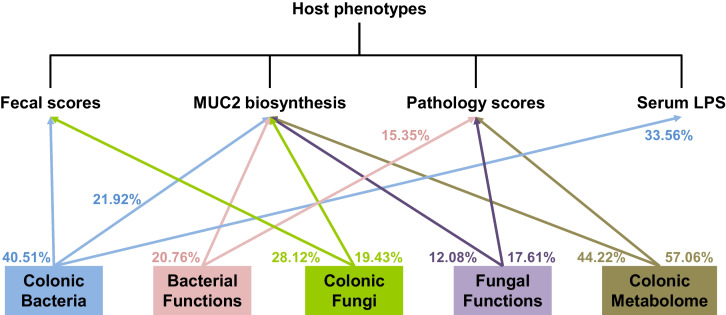
The proportion of variance in host phenotypes explained by the colonic microbiota, microbial functions, and metabolome. Only omics-based explainability ≥ 10% were displayed

Using linear mixed effect model (see “Methods”), the variation of phenotypes explained by the colonic microbiota, microbial functions, and metabolites are shown in Fig. 6. The variation of fecal scores explained by the colonic bacteria and fungi were 40.51 and 28.12%, respectively. The variation of MUC2 biosynthesis explained by the colonic bacteria, bacterial functions, fungi, fungal functions, and metabolites were 21.92, 20.76, 19.43, 12.08, and 44.22%, respectively. The variation of pathology scores explained by the colonic bacterial functions, fungal functions, and metabolites were 15.35, 17.61, and 57.06, respectively. The variation of serum LPS explained by the colonic bacteria was 33.56%.

## Discussion

Colon plays an important role in energy metabolism and intestinal health of ruminants during early life and other physiological phases [13, 14, 20, 53, 53]. By using a high-grain or high-starch diet, colonic inflammation has been linked to an altered microbiota [13, 53, 53], but the possibility that colonic dysbiosis may be derived from the potential effects of a high-starch diet on ruminal development and fermentation disorders has not been excluded. By maintaining the same dietary starch content and changing only the corn processing methods, which simultaneously changed the rate of starch digestion in the rumen and passed through the rumen [53], the HES diet with crushed corn increased the hindgut starch content but did not significantly change the ruminal fermentation indices, development, or health conditions compared with LES diets with whole corn. Hence, the identified colonic inflammation was mainly derived from colonic microbiota disorders and may not be affected by ruminal dysfunction. Notably, our study is one of the few studies that demonstrated altered colonic microbiome, metabolome, and host transcriptome in response to HES feeding and its associated pathologies in young ruminants. Metagenomics sequencing showed the dynamic changes of colonic microbiome in the goats and found the impacts of colonic microbial dysbiosis in response to different enteral starch diets. The observed significant shifts in host-microbiome interactions led to impaired mucosa and systemic inflammation.

First, our study revealed that changes in the microbiome at both taxonomic and functional levels were associated with decreased colonic MUC2 expression in response to HES feeding. Lower MUC2 expression indicated the depletion of major structural mucus components or reduction of the response of goblet cells to microbial challenge, abnormal penetrability, and breakdown of intestinal barrier function induces colonic inflammation [53, 53]. Generally, *Muribaculaceae*, *Bifidobacterium*, and *Bacteroidales* can degrade all or most O-glycan mucosal sugars [53]. But in the present study, no microbial association with colonic MUC2 expression was found. The analysis of omics basing explainability of phenotype variations showed that the variation of MUC2 biosynthesis explained by the colonic bacteria, bacterial functions, fungi, fungal functions, and metabolites were 21.92, 20.76, 19.43, 12.08, and 44.22%, respectively. Thus exhibits a comprehensive effect of multiple factors in contributing or positively interacting with other microorganisms responsible for colonic MUC2 depletion. Previous studies showed that the DCA and CDCA breakdown the epithelial tight junctions and contribute to inflammatory bowel disease (IBD) and irritable bowel syndrome (IBS) [53, 53, 53, 53]. The DCA and CDCA were enriched in HES group, implying that the presence of higher level of DCA and CDCA increases the risk of developing mucosal barrier dysfunction and inflammation. Together with the higher serum LPS in HES goats, it suggests that enteric starch increased mucosal permeability by selectively increasing the abundance of bacteria with enhanced mucin degradation capacity.

Our study further focused on the roles of altered microbiome in affecting colonic mucosal immunologic state between LES and HES goats. First, overall analyzed the key differential genes, *TRAV* and *c-Maf*, that separately severing as an antigen receptor of CD4^+^/8^+^ T cells or the promoter of the T_H_2 cell maturation [53], were linked to upregulated antigen processing and presentation and inflammatory bowel disease (IBD) pathways in colonic mucosa of HES goats. Further, since the IL-6 activates both T_H_1 and T_H_2 cell continually during inflammation, the role of T_H_1/T_H_2 polarization in mucosal immunity was then tested. IL-12 induces T_H_1 cell differentiation by activation and phosphorylation of the signal transducer and activator of transcription 4 (STAT4), and drives T_H_1-mediated intestinal inflammation [53]. T-bet regulates the IFN-γ production in T_H_1 cells, and both IFN-γ and T-bet will result in T_H_1-mediated intestinal immune disorder [53]. However, these key factors were not significantly changed between LES and HES goats, which indicated that HES-induced colonic inflammation in young goats may not be caused by T_H_1-mediated mucosal immune disorders. As the master transcription factors, *c-Maf* and *GATA3* are only expressed in T_H_2 cell which improves the production of *IL-4*, *IL-5*, and *IL-13* in T_H_2 cell [53]. IL-5 and IL-13 correlate with T_H_2 polarization and T_H_2-mediated ulcerative colitis [53, 53]. Early study showed that IL-13 was associated with epithelial cell apoptosis and increased expression of the Claudin2 [53]. In the current study, upregulation of *c-Maf*, *GATA3*,* IL-5*, and *IL-13* in the colonic mucosa of HES goats confirmed that the colonic inflammation in growing goats caused by HES was attributed to the T_H_2 cytokine pattern, which might be related to the T_H_2-mediated immune disorder. On this basis, colon microbiome and their metabolome in affecting colonic mucosal immunologic state were studied in the present study. The *Bacteroides sp. CAG:927, Prevotella sp. P4-67*, and *Prevotella sp. PINT* were enriched in HES group. *Bacteroides sp. CAG:927* can cause endogenous infection during the disruption of intestinal microbiota. *Prevotella* is saccharolytic or moderately saccharolytic that promotes the production of acetate, succinate, and a few isobutyrate, isovalerate, and lactate [53]. As an opportunistic pathogen, *Prevotella* has been proven to infect the host with other microorganisms when the ecological conditions change [53]. Previous study showed that the colonization of the *Prevotella* and *Muribaculaceae* could exacerbate intestinal inflammation [53]. Our study demonstrated that *Bacteroides sp. CAG:927*, 2 species of *Prevotella* (*Prevotella sp. PINT* and *Prevotella sp. P4-67*), and 6 differential bacterial functions (ko04215, ko04660, ko04664, ko05170, ko05210, and ko05323) were closely related to the expression of immune-related DEGs that includes *TRAV*, *KIR3DL*, and *c-MAF* for antigen presentation; *c-MAF*, *GATA3*, *IL-5*, and *IL-13* for T_H_2-mediated inflammatory process; *A1AT1* and *C3* for complement and coagulation cascades, indicating that the T_H_2-mediated colonic inflammation of the HES goats could be attributed to these bacterial species and functions [53].

Besides, the present study also revealed the roles of colonic fungal community and fungal functions in affecting the colonic mucosal immunologic state differences between LES and HES goats. Our study showed that the *Hypoxylon sp. EC38*, *Fusarium vanettenii*, *Fusarium sp. AF-8*, *Neocallimastix californiae*, and fungal functions (ko05203, ko05034, and ko05322) had positive correlations with immune-related DEGs that includes *TRAV*, *KIR3DL*, and *c-MAF* for antigen presentation; *c-MAF*, *GATA3*, and *IL-5* for T_H_2-mediated inflammatory process; as well as *A1AT1* and *C3* for complement and coagulation cascades. Of these fungi, *Fusarium vanettenii* and *Fusarium sp. AF-8* both belong to the genus of Fusarium, which was widely suggested to produce the Fusarium mycotoxins that includes the Zearalenone [53]. Hence, the increased potential pathogenic roles of these fungi under the HES diet may be attributed to their produced Fusarium mycotoxins, which were also widely suggested to induce the gut inflammation through activating T_H_2-mediated inflammatory process [53, 53].

Last, in the present study, we evaluated the proportions of variation in host phenotypes that explained by colonic microbiota, microbial functions, and luminal metabolome[53]. The contributions of the colonic bacteria and bacterial functions were higher than the fungi and fungal functions on the fecal scores and MUC2, indicating that the colonic bacteria occupied a dominant position in the connection between environmental factors and host responses in the colon. The omics-explainability of the colonic luminal metabolome in our study suggests that the colonic metabolism makes greater contributions to MUC2 and pathologic scores compared with the colonic microbiota and functions [53]. Therefore, fungi and metabolites should be incorporated into the future systemic studies aimed at hindgut dysbiosis in young ruminants. Our data could help in advancing our understanding of the interactions between the microbiome and host in the hindgut of young ruminants. However, we acknowledge that some limitations should be noted. The present study exhibits that the HES diet had a comprehensive effect of multiple factors in mediating colonic inflammation of goats. Investigating the causality of HES diet and hindgut dysbiosis should be continually conducted in the future even though our findings cannot be evidence of causation. Further systematic research, e.g., metagenome assembled genomes, proteome, or other multi-omics research, is needed to complement our findings due to the few studies that have investigated the influence of hindgut homeostasis on intestinal functions and health in young ruminants.

## Conclusions

**Fig. 7:**
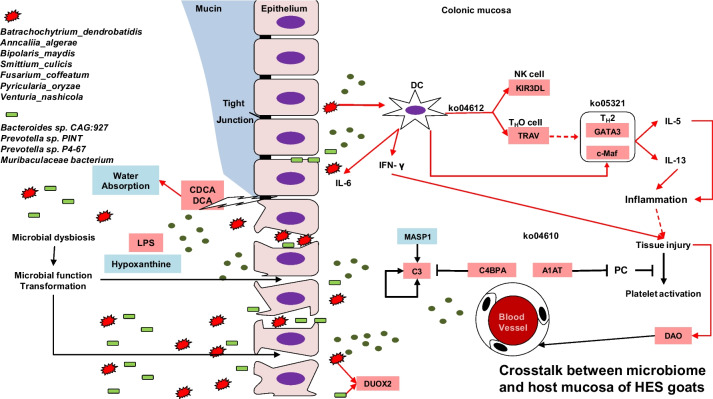
Crosstalk between microbiome and host mucosa in enteral starch content-associated colonic inflammation of HES goats. HES diet caused increased starch content and concentrations of BAs in the colon and inhibited host mucosal MUC2 expression and epithelia tight junction, weakening the epithelia barrier. Colonic microbiome and its metabolites stimulated T_H_2-mediated inflammatory process in colonic mucosa. Red rectangle, upregulation; blue rectangle, downregulation. Red arrow, increase; blue arrow, decrease. Abbreviations: DUOX2, dual oxidase 2; DAO, diamine oxidase; LPS, Lipopolysaccharide; *TRAV*, T cell receptor alpha chain V region; *c-Maf*, Transcription factor Maf; *C4BPA*, Complement component 4 binding protein alpha; CDCA, chenodeoxycholic acid; DCA, deoxycholic acid; *GATA3*, master transcription factor. ko04612, Antigen processing and presentation; ko04610, Complement and coagulation cascades; ko05321, Inflammatory bowel disease (IBD)

This is one of the most comprehensive studies with the aim of elucidating the molecular mechanisms behind the colonic dysbiosis in response to HES diet. In summary, our study demonstrated that HES caused BA accumulation, and weakened host mucosal MUC2 biosynthesis and epithelial tight junction, resulting in luminal macromolecule breaching the physical barrier. Colonic microbiome and its metabolites stimulated colonic inflammation and tissue injury *via* promoting the antigen presentation and promoting T_H_2-mediated inflammatory process. Furthermore, the capacity of colonic water absorption was also repressed (Fig. 7). The present study provides systemic insight into the disrupted colonic homeostasis in a goat model. The nonnegligible contribution of fungi is worth more future studies in hindgut microbe-host interactions. It will be instrumental in developing new nutritional strategies to relieve the overload starch-mediated gut dysfunction and hindgut dysbiosis in young ruminants.

### Supplementary Information

**Additional file 1: Table S1.** Ingredient and chemical composition of the basal diet. **Table S2.** The specific primers for the qPCR of *β-Actin* and tested mRNAs. **Table S3.** Effects of different hindgut enteral starch diets on the rumen fermentation parameters in growing goats. **Table S4.** The origin of metabolites. **Figure S1.** The flow chart of the present study. **Figure S2.** Fecal evaluation system for dairy goats. **Figure S3.** The dry matter intake (DMI) and content of luminal nutrients (*n* = 20 for DMI, and *n* = 8 for luminal content of nutrients). **Figure S4.** The gene expression of colonic bile acids receptors (*n* = 6). **Figure S5.** Spearman correlation among the host phenotypes, nutrients, microbes, and microbial functions.

## Data Availability

The colonic transcriptome raw files and digesta microbiome sequencing reads are available in the Sequence Read Archive (SRA) of NCBI under accession project number: PRJNA737607 and PRJNA836944, respectively. The colonic metabolome data have been deposited in the OMIX of China National Center for Bioinformation/Beijing Institute of Genomics, Chinese Academy of Sciences (CNCB) under accession project number: OMIX005201.

## Abbreviations

BW: Body weight
DMI: Dry matter intake
NDF: Neutral detergent fiber
ADF: Acid detergent fiber
MUC2: Mucin-2
DUOX2: Dual oxidase 2
DAO: Diamine oxidase
LPS: Lipopolysaccharide
AQPs: Aquaporin
NHE: Na^+^/H^+^ exchanger
CFTR: Cystic fibrosis transmembrane conductance regulator
ClCN2: Voltage-gated chloride channel 2
TRAV: T cell receptor alpha chain V region
c-Maf: Transcription factor Maf
A1AT: Alpha-1-antitrypsin
CDH26: Cadherin26
VSIG1: V-set and immunoglobulin domain containing 1
MASP1: Mannan-binding lectin serine protease 1
KIR3DL: Killer cell immunoglobulin-like receptor 3DL
C4BPA: Complement component 4 binding protein alpha
CLDN4: Claudin 4
CMIP: C-Maf Inducing Protein
T-bet: T-box transcription factor 21
XDH: Xanthine dehydrogenase
PNP: Purine nucleoside phosphorylase
CDCA: Chenodeoxycholic acid
DCA: Deoxycholic acid
BAs: Bile acids
