# Supplementary material for: Multi-omics reveal mechanisms of high enteral starch diet mediated colonic dysbiosis via microbiome-host interactions in young ruminant
Source: Microbiome. 2024 Feb 24;12:38. doi: 10.1186/s40168-024-01760-w (PMC10893732; doi:10.1186/s40168-024-01760-w)
Supplement: Supplementary file 2 — Additional file 1: Table S1. Ingredient and chemical composition of the basal diet. Table S2. The specific primers for the qPCR of β-Actin and tested mRNAs. Table S3. Effects of different hindgut enteral starch diets on the rumen fermentation parameters in growing goats. Table S4. The origin of metabolites. Figure S1. The flow chart of the present study. Figure S2. Fecal evaluation system for dairy goats. Figure S3. The dry matter intake (DMI) and content of luminal nutrients (n = 20 for DMI, and n = 8 for luminal content of nutrients). Figure S4. The gene expression of colonic bile acids receptors (n = 6). Figure S5. Spearman correlation among the host phenotypes, nutrients, microbes, and microbial functions. [file 40168_2024_1760_MOESM1_ESM.zip › Supplementary materials second revison.docx]

Multi-Omics Reveal Mechanisms of High Enteral Starch Diet Mediated Colonic Dysbiosis *via* Microbiome-Host Interactions in Young Ruminant

Chunjia Jin^1,3, #^, Shengru Wu^1, 3, #, *^, Ziqi Liang^1,3^, Jun Zhang^1,3^, Xinjian Lei^1,3^, Hanxun Bai^1,3^, Gaofeng Liang^1,3^, Xiaodong Su^1,3^, Xiaodong Chen^1,3^, Peiyue Wang^1,3^, Yue Wang^1,3^, Leluo Guan^2, *^, Junhu Yao^1,3, *^

**Author affiliations:**

^1^ College of Animal Science and Technology, Northwest A&F University, Yangling, Shaanxi 712100, P.R. China

^2^ Department of Agricultural, Food and Nutritional Science, University of Alberta, 116 St. and 85 Ave., Edmonton, Alberta, T6G 2P5, Canada

^3^ Key Laboratory of Livestock Biology, Northwest A&F University, Yangling 712100, Shaanxi, China

# These authors contributed equally to the present study and shared the first authorship.

**^*^Co-correspondence:**

E-mail: [yaojunhu2004@sohu.com](mailto:yaojunhu2004@sohu.com) (JH Yao); [leluo.guan@ualberta.ca](mailto:leluo.guan@ualberta.ca) (LL Guan); wushengru2013@163.com (SR Wu)

**Supplementary files**

Table S1. Ingredient and chemical composition of the basal diet.

Table S2. The specific primers for the qPCR of *β-Actin* and tested mRNAs.

Table S3. Effects of different hindgut enteral starch diets on the rumen fermentation parameters in growing goats.

Table S4. The origin of metabolites.

Figure S1. The flow chart of the present study.

Figure S2. Fecal evaluation system for dairy goats.

Figure S3. The dry matter intake (DMI) and content of luminal nutrients (n=20 for DMI, and n=8 for luminal content of nutrients).

Figure S4. The gene expression of colonic bile acids receptors (n=6).

Figure S5. Spearman correlation among the host phenotypes, nutrients, microbes, and microbial functions.

| Table S1. Ingredient and chemical composition of the basal diet | | |
| --- | --- | --- |
| Item | Treatment diet | |
|  | LES | HES |
| Ingredients, % DM |  |  |
| Whole corn | 20.9 | - |
| Crushed corn | - | 20.9 |
| Corn silage | 20.6 | 20.6 |
| Alfalfa hay | 39.9 | 39.9 |
| Wheat bran | 10.7 | 10.7 |
| Soybean meal | 5.9 | 5.9 |
| Premix^1^ | 0.1 | 0.1 |
| CaHPO_4_ | 0.3 | 0.3 |
| Salt | 1.3 | 1.3 |
| NaHCO_3_ | 0.3 | 0.3 |
| Chemical composition (% of Dry matter)^2^ | |  |
| Dry matter | 57.40 | 57.40 |
| Crude protein, CP | 15.77 | 15.56 |
| Neutral detergent fiber, NDF | 30.35 | 30.45 |
| Acid detergent fiber, ADF | 16.04 | 15.61 |
| Starch | 23.53 | 24.68 |

DM=dry matter.

^1^The premix contain (per kilogram): 2925 mg of Cu (as CuSO_4_·5H_2_O), 3900 mg of Fe (as FeSO_4_·H_2_O), 2750 mg of Zn (as ZnSO_4_·H_2_O), 800 mg of Mn (as MnSO_4_·H_2_O).

^2^ Chemical compositions are the measured value.

| Table S2. The specific primers for the qPCR of *β-Actin* and tested mRNAs | | | |
| --- | --- | --- | --- |
| Gene | Accession number | Product size, bp | Primer sequence (5′-3′) |
| *Claudin1* | XM_005675123.3 | 105 | F: GCTGCTGCTTCTCTCTGCCTTC |
|  |  |  | R: AGGTGCTGGCTTGGGATAGGG |
| *Claudin4* | XM_005697785.2 | 110 | F: TCATCGGCAGCAACATCGTCAC |
|  |  |  | R: CAGCAGCGAGTCGTACACCTTG |
| *Occludin* | XM_018065681.1 | 105 | F: GTGGTAACTTGGAGACGCTTTC |
|  |  |  | R: CTCCCGTCGTGTAGTCTGTT |
| *ZO-1* | XM_018066118.1 | 84 | F: ACCACACTGTGATCCTAAAACCT |
|  |  |  | R: ACAGAAACACAGTTTGCGCC |
| *IL-1B* | XM_013967700.2 | 99 | F: TCCACCTCCTCTCACAGGAAA |
|  |  |  | R: TACCCAAGGCCACAGGAATCT |
| *IL-2* | NM_001287567.1 | 115 | F: AACGGTGCACCTACTTCAAGC |
|  |  |  | R: AGAGCTTGAGGTTCTCGGGAT |
| *IL-6* | NM_001285640.1 | 122 | F: ACCACTCCAGCCACAAACAC |
|  |  |  | R: CTCAGGCTGAACTGCAGGAA |
| *IL-10* | XM_005690416.3 | 119 | F: GTCTGGTGCAGTGAGCTTCT |
|  |  |  | R: CTCGTGGATCCAGACGTAGC |
| *IL-12* | NM_001285700.1 | 90 | F: GTCCTCACGTGTGACACTCC |
|  |  |  | R: GGTCAAGGTTTTGCCAGAGC |
| *IL-22* | NM_001285758.1 | 131 | F: GCCTCCTTGTCATGGTGCTGTG |
|  |  |  | R: AGCCTCCTGAGCCAGAGTGAAG |
| *IFN-γ* | NM_001285682.1 | 82 | F: CGGCAGCTCTGAGAAACTGGAG |
|  |  |  | R: TATGGCTTTGCGCTGGATCTGC |
| *TNF-α* | NM_001286442.1 | 124 | F: GTGGAGCTGGCAGAGGAGGTG |
|  |  |  | R: GCAGGCAGAAGAGCGTGGTG |
| *β-Actin* | NM_001314342.1 | 92 | F: GGCACCCAGCACGATGAAGATC |
|  |  |  | R: TGGACAGCGAGGCCAGGATG |
| *AQP1* | XM_018047113.1 | 91 | F: GACACCTGCTGGCGATAGAC |
|  |  |  | R: TGGTCCTGGAAATTGTGCGT |
| *CFTR* | XM_013963305.2 | 126 | F: CCTTCTGGGAGGAGGGATTT |
|  |  |  | R: TCAGGACAGGAGTACCGAGG |
| *AQP3* | MN331772.1 | 113 | F: CGGTGGTTTCCTCACCATCA |
|  |  |  | R: ACATAGCAAAGGTCACGGCA |
| *CLCN2* | XM_013964033.2 | 89 | F: AAGCGGGTCCGAATTTCCTT |
|  |  |  | R: AGTTGCTGCTCTTCCCACTC |
| *CDH2* | XM_018039719.1 | 143 | F: TGTGATTCCAACGGGGACTG |
|  |  |  | R: TCCCGGCGTTTCATCCATAC |
| *CD74* | XM_018050159.1 | 81 | F: GGTTCCACCAAAAGACCCAA |
|  |  |  | R: AGGGCGACTGACTGAAATCC |
| *SERPINA14* | NM_001314301.1 | 100 | F: GCTTCCCAAGATTGACCCCA |
|  |  |  | R: TTGATGCAGGGCCTCCAAAT |
| *THBS4* | XM_018053668.1 | 97 | F: TGCAGACCATGAACAGCGAT |
|  |  |  | R: TCCGTCTGAGTGTTGACGTG |
| *COL28A1* | XM_005678961.3 | 94 | F: TATCTGGGGGAAGGCACGTA |
|  |  |  | R: CCAAGGCCACCTTTTTCACG |
| *VSIG1* | XM_005700219.3 | 108 | F: AGCGTCAGCGTGTTAGTCAA |
|  |  |  | R: AGGTGTTCCAAGCACCGAAA |
| *CDH26* | XM_005688418.3 | 124 | F: GACCGCGAATCTCCTCATGT |
|  |  |  | R: TGTCGTTGACGTCTGACAGG |
| *CLDN4* | XM_005697785.2 | 107 | F: CCGTCCCCCTTCTTCAATCC |
|  |  |  | R: TTCTCATGGCCCCAGGTTTC |
| *LAMA1* | XM_018039603.1 | 116 | F: AGCCTAACGCTCAACGTGAA |
|  |  |  | R: AGGAAGGCCACTTTTCCTCG |
| *KIR3DP1* | XM_018063120.1 | 143 | F: ACACCACAGAAGAAACATGGC |
|  |  |  | R: GGCAACATGATTTGAGGTAGAACAC |
| *IL-13* | XM_005682617.2 | 80 | F: TGGGGTTAGGAGGGACTGTT |
|  |  |  | R: CCCACTGCTTTAGTGCTGGA |
| *IL-4* | NM_001285681.1 | 113 | F: TCTGCAGGGCTGGAATTGAG |
|  |  |  | R: AGAACAGGTCTTGCTTGCCA |
| *TRAV* | L18956.1 | 84 | F: GGCTCTACCTGATTGTCCCC |
|  |  |  | R: GACCCTGTACCCAGGTGACT |
| *IL-5* | XM_005682619.2 | 96 | F: GCTCTTGGAGCTGCCTATGT |
|  |  |  | R: AGTTTGATGCGTGGAGAGCA |
| *GATA3* | XM_018056969.1 | 93 | F: TTTGCAGGCATCAAGCAACC |
|  |  |  | R: ATGGGATCCAGATTCGGGGA |
| *NRL* | XM_018054615.1 | 96 | F: CGCCTGTGCCATTCACTAGA |
|  |  |  | R: GAGGGTCCAGGAAGTTTGGG |
| *CMIP* | XM_018061764.1 | 143 | F: CAGTTCGTCTCCCGCGAAG |
|  |  |  | R: CAACGGCTCGGGTTACTCA |
| *SPOCK1* | XM_018050345.1 | 137 | F: ACCGCTTTCGAGACGAAGTT |
|  |  |  | R: CTGGGTCACACACACCTTGT |
| *MASP1* | XM_005675141.3 | 115 | F: CCAAACCTGGTCAAGCGGAT |
|  |  |  | R: CGAACCACTTGTCATTGGGC |
| *C4BPA* | XM_013970043.2 | 88 | F: ACCTGCAGTCCCTCAGTGTA |
|  |  |  | R: AGCACTTTATCAGACTTTGGGGT |
| *TBX21* | XM_018064958.1 | 105 | F: GAGGTGTCGTTCGGGAAACT |
|  |  |  | R: CAGCCGGGGCTGGTATTTAT |
| *c-Maf* | XM_018061746.1 | 95 | F: GATGCTGGCATGCTAATCGC |
|  |  |  | R: ACACCATTCGCGTGTCTTCT |
| *NHE2* | XM_018054964.1 | 85 | F: AGATCCGTCAGCGAACCTTG |
|  |  |  | R: CGGATCAGGATCTCCTTGGC |
| *NHE 3* | XM_018065649.1 | 110 | F: ACTGAGGAAGCCCCCAACTA |
|  |  |  | R: GCGGGGTTGTCAATTCCTGA |
| *NHE 1* | XM_018056627.1 | 140 | F: GAGATCCACACGCAGTTCCT |
|  |  |  | R: CGCTCACCAGCTATCAGACA |
| *C3* | XM_018050944.1 | 149 | F: TTCACGGATGCAGGACTCAC |
|  |  |  | R: CCGCTGTACTGACCCACTTT |
| *TFF3* | XM_005675635.3 | 83 | F: GCTTGTCTCACACTGCTCCT |
|  |  |  | R: GCTGAGCACGGGAGCTTTAT |
| *TGFB1* | NM_001314142.1 | 100 | F: ACGTGGAGCTGTACCAGAAAT |
|  |  |  | R: ACGTCAAAGGACAGCCACTC |
| *AQP8* | XM_018041074.1 | 106 | F: CTGGGGAATATCAGCGGTGG |
|  |  |  | R: GCTGGGAGATCCAGTAGGGA |
| *BMPER* | XM_005679197.2 | 144 | F: CGCAAGAAGCAGTGTGTTCC |
|  |  |  | R: TGTCCGTCCGTCAAAAGTGT |
| *DYNC1I1* | XM_018047394.1 | 108 | F: CCCTCTAGTCCCAACCCCTA |
|  |  |  | R: TGCAGGGTCCTTGTTAACGG |
| *PRTN3* | XM_018050500.1 | 81 | F: AGAACAACTACGACCCGCAG |
|  |  |  | R: CATGGGTGTTGAGGATGGCT |
| *TMPRSS6* | XM_018048301.1 | 80 | F: TCAGATCGTGAGTCCTGCCC |
|  |  |  | R: TCCTTGACCCCATCACAGGT |
| *TRPV5* | XM_018046845.1 | 148 | F: CGCCATCTGTGAGCCATTTG |
|  |  |  | R: GTTGTGAGGAGTGAGGCGAA |
| *XDH* | NM_001285624.1 | 106 | F: TGTACACGCTGCTTCGGAAT |
|  |  |  | R: CCCTGGAGGATGGGTCTGTA |
| *PNP* | XM_005685402.3 | 80 | F:CTCTGGCGCTGGGGATATAA |
|  |  |  | R: GGCTTCTGTCACCGAGTGTT |

| Table S3. Effects of different hindgut enteral starch diets on the rumen fermentation parameters in growing goats (n=8)^1^ | | | | |
| --- | --- | --- | --- | --- |
| Items | LES | HES | SEM | *P-*value |
| pH | 5.88 | 5.94 | 0.057 | 0.208 |
| Total SCFAs^2^, mM/L | 145.81 | 131.34 | 6.690 | 0.295 |
| Acetate, % | 64.53 | 66.15 | 0.752 | 0.300 |
| Propionate, % | 18.62 | 17.47 | 0.682 | 0.419 |
| Butyrate, % | 13.17 | 12.71 | 0.595 | 0.714 |
| Others, % | 2.83 | 2.71 | 0.077 | 0.454 |
| Acetate:Propionate | 3.39 | 3.75 | 0.320 | 0.280 |
| ^1^: The difference between two groups was identified by independent sample T-test. *P* < 0.05 indicated that mean values were significantly difference between the low hindgut-enteral starch content diet (LES) and the high hindgut-enteral starch content diet (HES).  ^2^: SCFA = short chain fatty acids | | | | |

Table S4. The origin of metabolites.

Notes: Detail table could be checked in another supplementary file with .xlsx format named “Table S4. The origin of metabolites.”


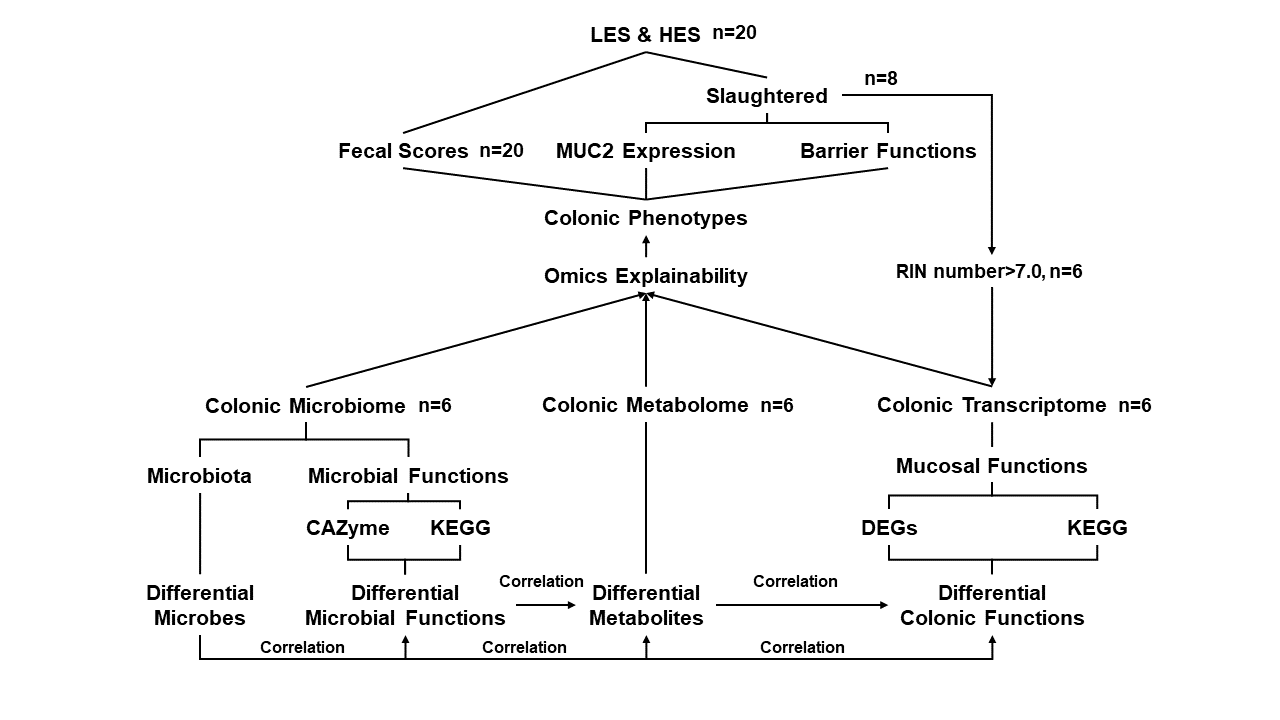


Figure S1. The flow chart of the present study


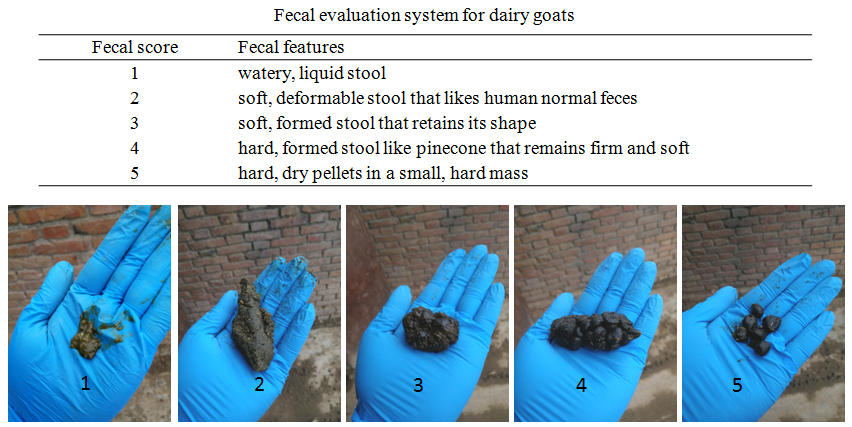


Figure S2. Fecal evaluation system for dairy goats.


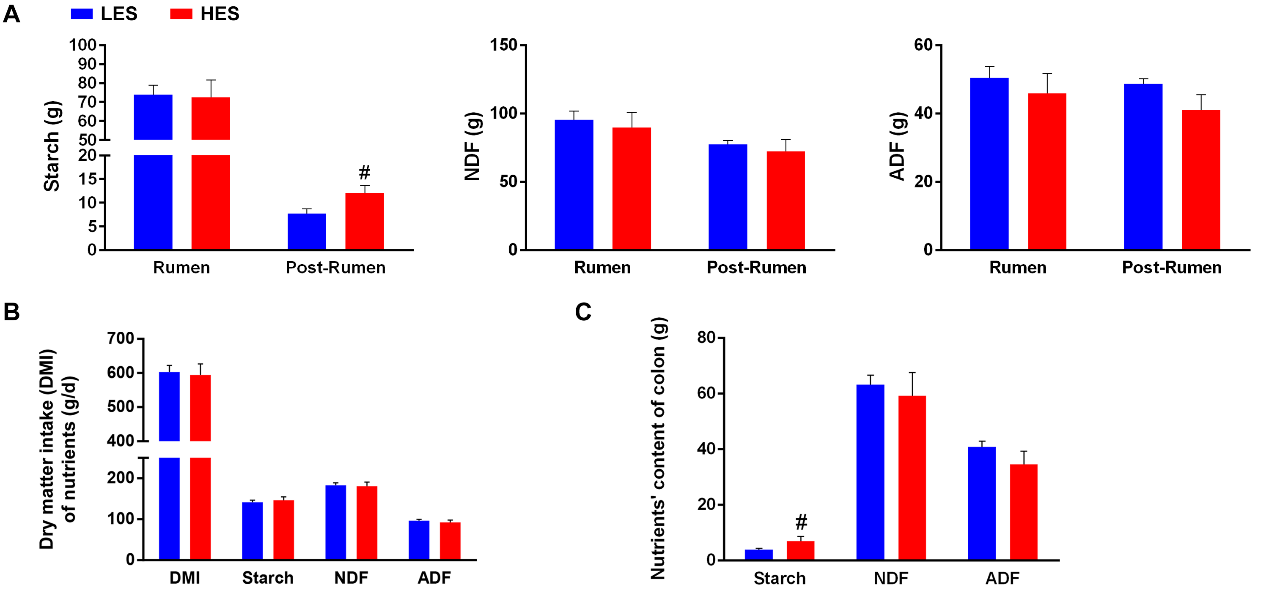


Figure S3. The dry matter intake (DMI) and content of luminal nutrients (n=20 for DMI, and n=8 for luminal content of nutrients).

(A) The content of rumen and rumen bypass nutrients of two groups (g).

(B) DMI of nutrients of two groups (g/d)

(C) Colonic nutrients content of two groups (g).

The difference between two groups was identified by independent sample T-test. Symbols indicate significance (#, 0.05 < *P* < 0.1).


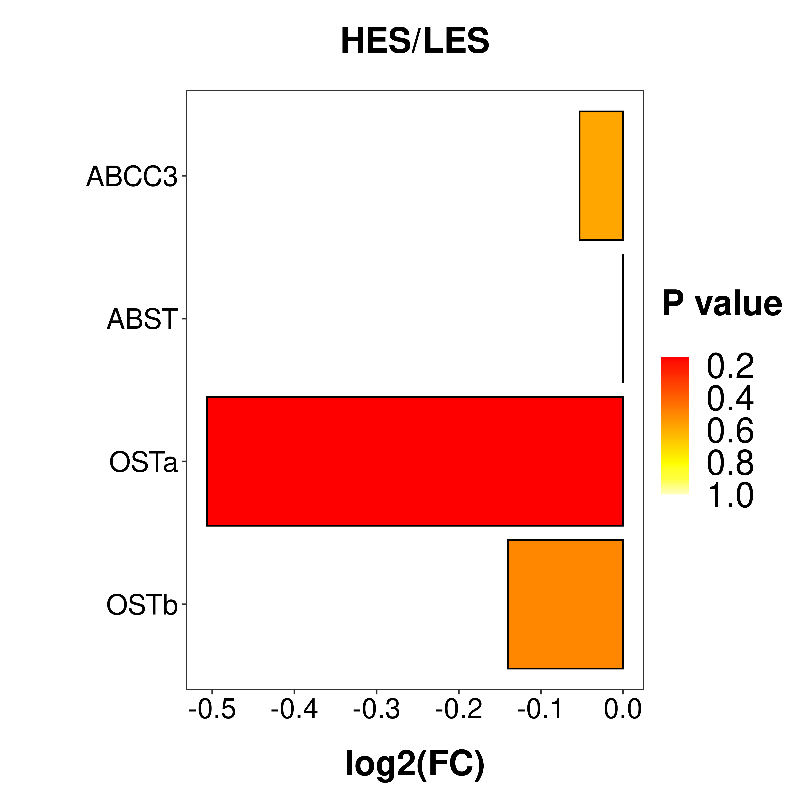


Figure S4. The gene expression of colonic bile acids receptors (n=6).


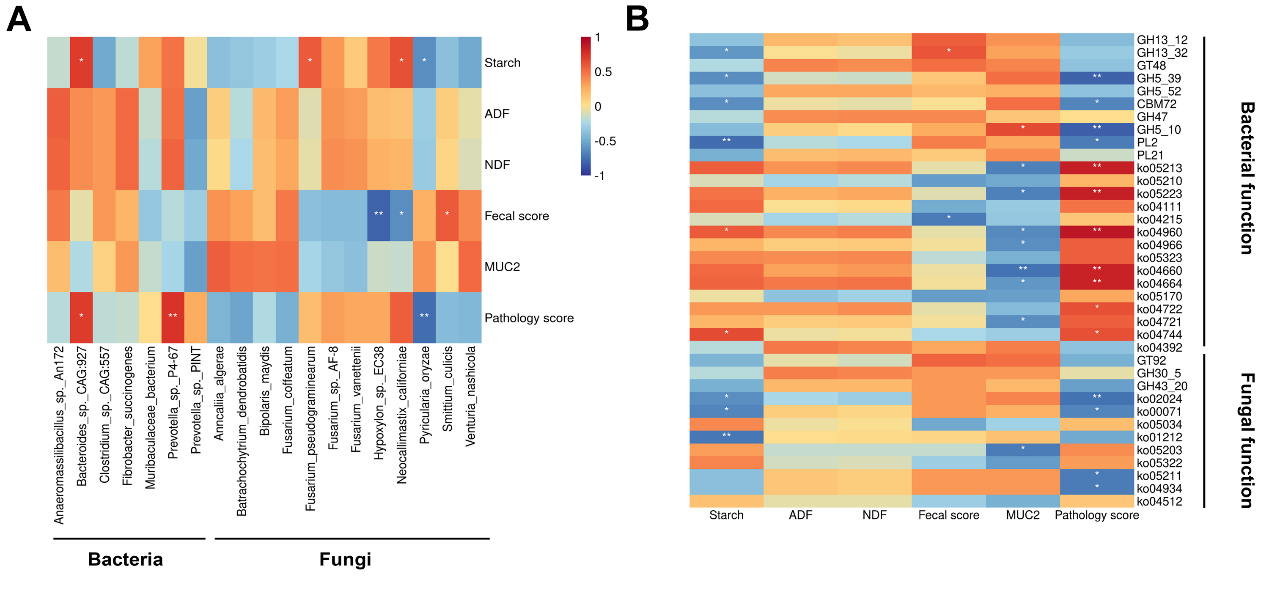


Figure S5. Spearman correlation among the host phenotypes, nutrients, microbes, and microbial functions.

(A) Heatmap of Spearman correlation among the host phenotypes, nutrients, and microbes.

(B) Heatmap of Spearman correlation among the host phenotypes, nutrients, and microbial functions.

ko05203, Viral carcinogenesis; ko05210, Colorectal cancer; ko05211, Renal cell carcinoma; ko05213, Endometrial cancer; ko05223, Non-small cell lung cancer; ko00620, Pyruvate metabolism; ko04111, Cell cycle - yeast; ko04215, Apoptosis - multiple species; ko02024, Quorum sensing; ko04934, Cushing syndrome; ko04960, Aldosterone-regulated sodium reabsorption; ko04966, Collecting duct acid secretion; ko01212, Fatty acid metabolism; ko05322, Systemic lupus erythematosus; ko05323, Rheumatoid arthritis; ko04660, T cell receptor signaling pathway; ko04664, Fc epsilon RI signaling pathway; ko05170, Human immunodeficiency virus 1 infection; ko00071, Fatty acid degradation; ko04721, Synaptic vesicle cycle; ko04722, Neurotrophin signaling pathway; ko04744, Phototransduction; ko04392, Hippo signaling pathway - multiple species; ko04512, ECM-receptor interaction; ko05034, Alcoholism.

Symbols indicate significance (**, |R|>0.5, *P*<0.01; *, |R|>0.5, *P*<0.05).
